# Risk of childhood mortality associated with death of a mother in low-and-middle-income countries: a systematic review and meta-analysis

**DOI:** 10.1186/s12889-019-7316-x

**Published:** 2019-10-11

**Authors:** Diep Thi Ngoc Nguyen, Suzanne Hughes, Sam Egger, D. Scott LaMontagne, Kate Simms, Phillip E. Castle, Karen Canfell

**Affiliations:** 1 Cancer Research Division, Cancer Council NSW, 153 Dowling Street, Woolloomooloo, Sydney, NSW 2011 Australia; 2 Prince of Wales Clinical School, Faculty of Medicine, UNSW, Sydney, Australia; 3 Department of Epidemiology & Population Health, Albert Einstein College of Medicine, New York, USA; 4 Centre for Vaccine Innovation and Access, PATH, Seattle, USA; 5 School of Public Health, Faculty of Medicine and Health, University of Sydney, Sydney, Australia

**Keywords:** Death of a mother, Childhood mortality, Low-and- middle-income country, Systematic review and meta-analysis

## Abstract

**Background:**

Death of a mother at an early age of the child may result in an increased risk of childhood mortality, especially in low-and-middle-income countries. This study aims to synthesize estimates of the association between a mother’s death and the risk of childhood mortality at different age ranges from birth to 18 years in these settings.

**Methods:**

Various MEDLINE databases, EMBASE, and Global Health databases were searched for population-based cohort and case-control studies published from 1980 to 2017. Studies were included if they reported the risk of childhood mortality for children whose mother had died relative to those whose mothers were alive. Random-effects meta-analyses were used to pool effect estimates, stratified by various exposures (child’s age when mother died, time since mother’s death) and outcomes (child’s age at risk of child death).

**Results:**

A total of 62 stratified risk estimates were extracted from 12 original studies. Childhood mortality was associated with child’s age at time of death of a mother and time since a mother’s death. For children whose mother died when they were **≤** 42 days, the relative risk (RR) of dying within the first 1–6 months of the child’s life was 35.5(95%CI:9.7–130.5, p [het] = 0.05) compared to children whose mother did not die; by 6–12 months this risk dropped to 2.8(95%CI:0.7–10.7). For children whose mother died when they were **≤** 1 year, the subsequent RR of dying in that year was 15.9(95%CI:2.2–116.1,p [het] = 0.02), compared to children whose mother lived. For children whose mother died when they were **≤** 5 years of age, the RR of dying before aged 12 was 4.1(95%CI:3.0–5.7),p [het] = 0.83. Mortality was also elevated in specific analysis  among children whose mother died when child was older than 42 days. Overall, for children whose mother died < 6 and 6+ months ago, RRs of dying before reaching adulthood (**≤**18 years) were 4.7(95%CI:2.6–8.7,p [het] = 0.2) and 2.1(95%CI:1.3–3.4,p [het] = 0.7), respectively, compared to children whose mother lived.

**Conclusions:**

There is evidence of an association between the death of a mother and childhood mortality in lower resource settings. These findings emphasize the critical importance of women in family outcomes and the importance of health care for women during the intrapartum and postpartum periods and throughout their child rearing years.

**Electronic supplementary material:**

The online version of this article (10.1186/s12889-019-7316-x) contains supplementary material, which is available to authorized users.

## Background

Women of reproductive age in low and middle income countries are more vulnerable to the both communicable and non-communicable diseases than those in high-income countries. Some of the leading causes of death in women globally, which are mostly among women in low-and-middle-income countries (LMICs), are death associated with childbirth, HIV/AIDS, tuberculosis (TB), injuries, cardiovascular disease, obesity, cervical cancer, violence, and depression and suicide [1]. Women in LMICs account for 99% of maternal death worldwide, up to 87% of global cervical cancer deaths, and suffer from the majority of cases of HIV/AIDS and TB globally [2–5]. The World Health Organization (WHO) has estimated that, over the period 1990–2015, there were substantial reductions in female mortality from tuberculosis (− 32%), HIV/AIDS (− 34%), and maternal mortality (− 47%) as a result of globally targeted intervention programs in low and middle income countries [2]. By contrast, there have been increases in mortality from other causes, including cervical cancer (+ 9%), road accidents (+ 36%) and suicide (+ 6%) in these countries [2].

Death of a mother has major consequences for children, as well as for families as a whole, given the important roles of mothers in taking care of family members and contributing to household income [6, 7]. The effect of a death of a mother on child health outcomes is more devastating when it happens in lower resource settings, where mothers are usually primary caregivers and social support is less widely available [8]. A number of studies have shown that loss of a mother in early childhood results in a substantial increase in the risk of childhood death, and that the magnitude of the risk of childhood mortality is associated with both age of child at death of a mother and the time since the death of a mother [9–11]. A recent systematic review and meta-analysis reported that the risk of child mortality at < 5 years associated with maternal death was 4·1 (95%CI: 2·4–7.0) [12]. This recent review provided the first overview on the association between death of a mother and early childhood mortality. However, the mortality risk of children aged < 5 years was estimated by combining the effect estimates/or frequencies reported for more finely stratified age groups. Both the age of the child at death of a mother and age at their own death could be important sources of heterogeneity in the association. Furthermore, the level of impact of death of a mother on childhood mortality could be modulated by many factors, including culture, socioeconomic status, disease burden, health care system, and social support networks, which differ between resource settings and time periods [13, 14]. A review focusing on lower income settings and considering stratified age groups, has potential to better elucidate the association in these settings.

This systematic review, therefore, aims to address the following research question: in LMICs (also referred to hereafter as lower resource settings), among the general population, for children whose mothers died during their childhood (varying age-intervals of ‘exposure’, up to 18 years of age), what is the risk of childhood mortality relative to those whose mothers were alive throughout the exposure period? In this review, the term “death of a mother” refers to a mother’s death from any cause, for children aged from birth up to a maximum of 18 years.

## Methods

The review has been undertaken in accordance with the PRISMA guidelines, and the PRISMA checklist for a report of a sysmatic review and meta-analysis has been used to complete this report (Additional file 4) [15].

### Selection criteria

Studies were included if they: were cohort or nested case-control study in a population of children under 18 years of age living in countries identified as low-income countries, lower-middle-income-countries, or upper-middle-income countries (according to the World Bank’s classification at the time the original studies were conducted) [16]; compared mortality risk for children according to maternal vital status and reported the effect measure as OR (Odds Ratio), RR (Rate Ratio/Risk Ratio/Relative Risk) or IRR (Incidence Rate Ratio), or HR (Hazard Ratio) using data collected after 1979; were published in English from 1/1/1980 to 31/3/2017. Studies were excluded if they: were editorials or reviews; reported pooled data from individual studies included in this review; or examined only HIV-infected mothers (children of an HIV+ mother are at higher risk of death [17]) or only pregnancy-related maternal deaths (as restricting to specific causes of mother’s death may introduce an additional source of bias). Papers that reported data collected before 1980 or used data from historical populations were excluded to ensure data are comparable and reliable among different studies. For meta-analyses, we included studies that reported any of the above-mentioned mortality-effect measures and refer to these effect measures as relative risks (Table S1A Inclusion and exclusion criteria and S1B List of excluded studies (Additional file 1)).

### Search strategy

We searched for relevant publications published during 1980–2017 on MEDLINE, MEDLINE in-process, Embase, and Global Health databases, which cover the international public health literature, including community health and epidemiology. A complete list of the search terms used for all search strategies is included in Table S2 Search strategy (Additional file 1). Initial searches of databases were completed in August 2016 and subsequently updated by monthly alerts until the end of March 2017. Reference lists of retrieved articles were examined and other websites, including Google, Google Scholar and the WHO website were searched for any additional relevant papers. We examined grey literature, for example annual reports on mortality from the Ministry of Health from countries where original studies were conducted (e.g. Bangladesh, South Africa). However, we found that these types of reports mainly reported the mortality rates of either women or children across different cohorts, whilst we required population-based studies which followed up a cohort of women and their children for a period of time and recorded their vital information during that period to estimate risk of childhood mortality associated with death of a mother. Titles and abstracts of the citations identified by the searches were scanned and clearly irrelevant articles and duplicates were excluded. The full texts of the remaining articles were retrieved for more detailed evaluation and assessment for inclusion against the selection criteria. Literature searching, citation screening and study selection were conducted by the first reviewer (DN) in consultation with the second, very experienced, systematic reviewer (SH). Any issues identified during the selection process by the first reviewer were raised and discussed with the second reviewer. All studies identified for inclusion by the first reviewer were independently assessed by the second reviewer for inclusion.

### Data extraction

The two reviewers agreed upon the data to be extracted and a specific data extraction format. For each included study, information was extracted on: (1) study characteristics - study design, setting, population, sample size, exposure, comparator, outcomes follow-up and statistical methods (2) results – risk estimates for exposed and non-exposed and effect measurement (OR, RR, IRR, HR) with 95% confidence interval (CI). If studies reported both the risk of death from all causes and the risk of death from all causes except HIV or tuberculosis, the risk estimates for death due to all causes except HIV or tuberculosis were extracted.

The study characteristics and results of all included studies were extracted independently by both reviewers (DN and SH). Disagreements were resolved by discussion between the two reviewers.

### Risk of bias assessment

Sources of bias were assessed independently by two reviewers using a 12-item risk of bias assessment tool developed specifically for cohort studies examining risk factors [18]. This tool was applied without adaptation and assesses the following potential sources of bias: selection of exposed and non-exposed populations, measurement of exposures, measurement of outcomes, timing of outcome with respect to exposure, adequacy of follow-up, participation rates, completeness of follow-up, accuracy of dates, differences in follow-up, differences in missing exposure data, control of confounding and appropriate inclusion of covariates in analyses.

For the assessment of confounding control, age of the child, the family socioeconomic status, the mother’s education and HIV status in areas of high HIV prevalence, were pre-specified as important confounders. Where there were several different sets of data published for the same cohort, each set of data was assessed.

### Summary measure, statistical analysis and data synthesis

Initial review of selected studies showed that the risks of childhood mortality varied according to exposures (age of child at death of a mother and time since death of a mother) and outcome (age at risk of child death). Thus, pooled analyses were stratified by both exposures and outcome. The study exposures were ‘death of a mother’ by child age when mother died (15 papers from 12 studies reported various child age ranges at death of a mother: “0–42 days”, “0–12 months”, “0–5 years”, and “0–10 years”) and ‘death of a mother’ by time since mother died (2 studies reported two time periods since mother died: “<6 months ago” and “≥6 months ago”). Various outcome age-intervals were reported, including: early neonatal (0–7 days), late neonatal (8–28 days), neonatal (0–28 days), post-neonatal (1–6 months; 6–12 months), infant (0–1 year), under-five (0–5 years) and other various age ranges (1–2 years; 2–3 years; 3–4 years; 1–5 years, 5–10 years, 0–10 years, and 0–18 years).

Given all included studies were cohort studies conducted in LMICs from 1980s onwards, random-effects meta-analysis techniques were used to pool effect estimates of the within-strata results reported by individual papers, using STATA version 13.1 [19]. In forest plot representations, summarystatistics below “mother died” and “mother survived” columns were included for descriptive purposes and were not used to calculate the adjusted effect estimates (which were extracted directly from the study reports). Overall effect estimates of different age strata were not pooled in meta-analyses that included more than one estimate from the same cohort. Additionally, as age of child is a potential confounder, we decided to simply include the extracted effect estimates as reported without combining among different age groups. Because overall death is quite a rare endpoint in children age 0–18, ORs, RRs, IRRs and HRs should be reasonable approximations of each other and are referred to hereafter simply as relative risks (RRs) [20–22].

Heterogeneity was assessed using the Chi^2^ test statistic and the I^2^ statistic [15]. Heterogeneity was considered significant if *p* < 0.05 for the Chi^2^ test statistic and where significant and data permitting for sensitivity analysis, potential sources of heterogeneity were explored. Potential sources of heterogeneity considered were HIV prevalence and risk estimate metrics. We were unable to assess publication bias or small-study effects in this review because of the limited number of studies within homologous outcome/exposure strata (and we did not assess a funnel plot of all effect estimates together because heterogeneity due to variation in the underlying effects being measured is known to induce spurious funnel plot asymmetry) [23].

## Results

### Study selection

**Fig. 1:**
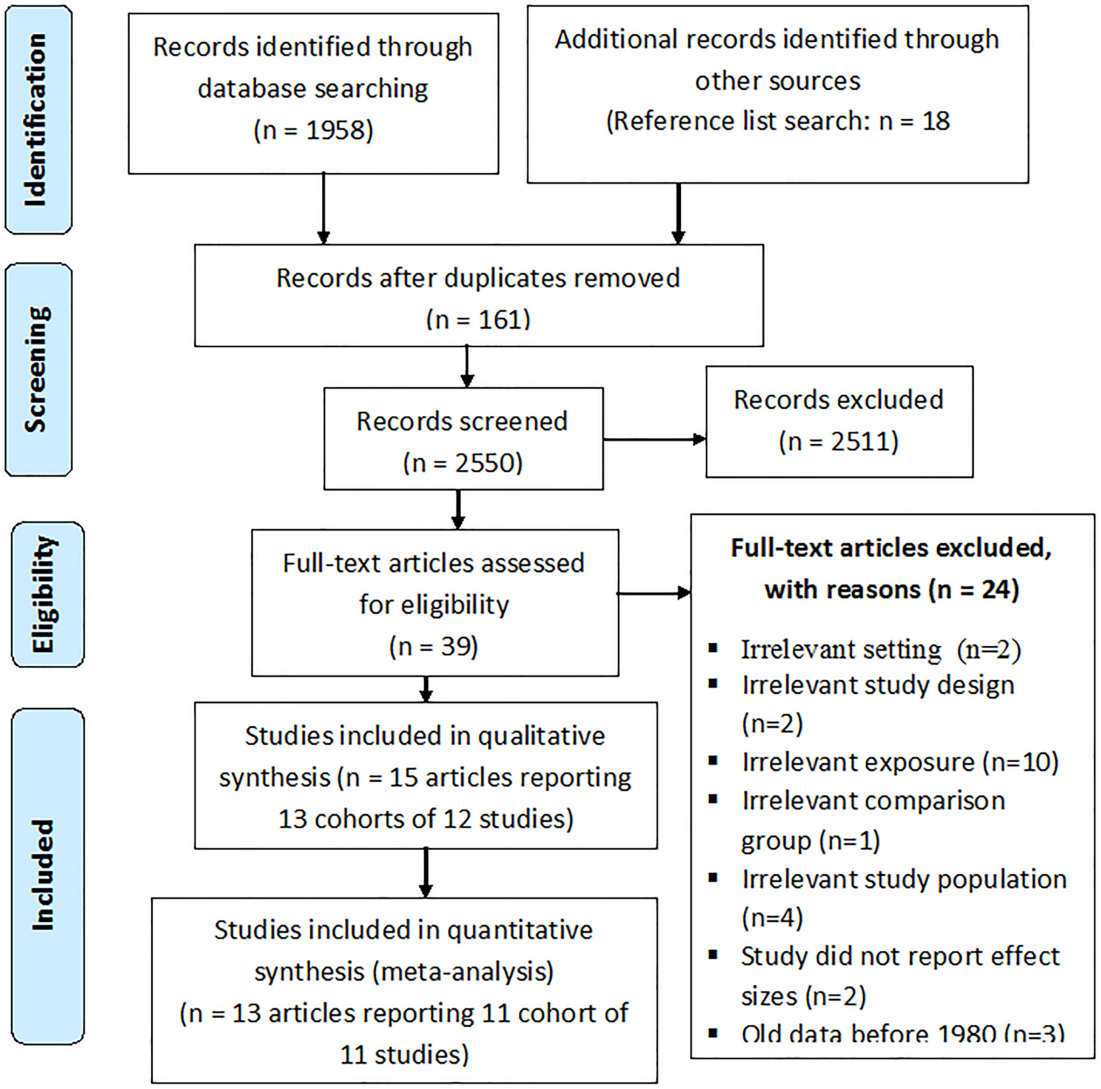
Flow diagram of study selection. Note: Flow diagram follows PRISMA

We identified 1958 citations from by literature search, 18 articles via reference searches and hand-searching of other sources, and 735 articles from monthly alerts (Fig. 1). After screening for titles and abstracts, 39 full text papers were retrieved and assessed for inclusion against the selection criteria. Included in this systematic review were 13 cohorts of 12 original studies, published in 15 articles, reporting 16 sets of data; 4 different data sets were reported for one cohort in four different papers [11, 24–26] and results for 2 cohorts were reported in one article [27]. Reasons for exclusion for 24 studies were as follows: conducted in a developed country (*n* = 2), data collected before 1980 (*n* = 3), study design (*n* = 2), not relevant exposure (*n* = 10), irrelevant comparison groups (*n* = 1), ineligible study population – all HIV (+) mothers (*n* = 4), and no effect size reported (*n* = 2). For meta-analysis, we included 13 articles reporting 11 cohorts of 11 original studies. Houle et al. [26] and Anderson et al. [28] were not included in the meta-analyses as they did not report results for the exposures of child’s age at the time of mother’s death or time since mother’s death.

### Study characteristics

The main characteristics of the 15 articles reporting 13 cohorts of 12 original studies included in qualitative review, of which 13 articles reporting 11 cohorts of 11 original studies were included in meta-analysis, are presented in Table 1 and Additional file 2: Tables S5 and S6. Of the total 12 original studies, 8 studies were conducted in Africa (Burkina Faso, Tanzania, Guinea-Bissau, Ethiopia, Uganda, Gambia, and South Africa), 2 studies were conducted in Asia (Bangladesh, Nepal), 1 study was conducted in the Caribe (Haiti), and the last study was conducted in 6 different countries (Argentina, Guatemala, India, Kenya, Pakistan and Zambia). Of a total 13 cohorts, 11 cohorts resided in rural and urban settings in low-income countries (Masmas et al., 2004 reported 2 cohorts) [10, 27–33, 35]; one cohort resided in a rural area of an upper-middle-income country [11, 24–26]; and the pooled cohort resided in rural and semi urban areas of low-income countries, lower-middle income countries, and an upper-middle income country [34]. Nine articles were published between 2010 and 2017; the remainder were published between 2003 and 2007. Demographic surveillance systems were used to collect data for nine of the cohorts.

**Table 1:** Summary of 15 articles reporting 13 cohorts of 12 original studies included in systematic review, 1980–2017

First author, year of publication	Country	Study setting	Data collection years	Study design	Sample size	Number of deaths	Exposure	Outcomes	Effect size (95%CI)
Anderson, 2007 [28]	Haiti	Rural		Nested case-control	167	13	Mother died during a pregnancy or within 42 days of a delivery	Child mortality at ages 0–12 years	OR = 1.06 (0.94–1.08)^k^
Becher, 2004 [29]	Burkina Faso	Rural	1993–1999	Cohort	10,222	1340	Mother died when child was 0–35 months	Child mortality at ages: 0–1 year 1–5 years	RR = 15.6 (7.61–31.8)^b^ for 0–1 year RR = 5.35 (1.69–16.9)^b^ for 1–5 years
Clark, 2013 [11]^r^	South Africa	Rural	1994–2008	Cohort	41,584	1244	Mother died when child was 0–5 years	Child mortality at age 0–5 years	OR = 3.93 (2.30–6.72)^e^
							Mother died 0–2 months ago	Child mortality at age 0–5 years	OR = 7.01 (3.16–15.56)^d^
							Mother died 3–5 months ago	Child mortality at age 3 months-5 years	OR = 4.03 (1.53–10.58)^d^
							Mother died 6–60 months ago	Child mortality at age 6 months-5 years	OR = 1.59 (0.61–4.15)^d^
Houle, 2015 [26]^r^			1992–2013		70,418	1747	Mother died within 43–365 days of most recent birth	Child mortality at 43 days-10 years	RR = 12.45 (4.74–32.70)^c^
							Mother died more than 1 year after most recent birth	Child mortality at 1–10 years	RR = 10.36 (4.77–22.48)^c^
Sartorius, 2010 [24]^r^			1992–2007		31,804	737	Mother died when child was 0–1 year	Child mortality at age 0–1 year	IRR = 51.1 (8.5–200.8)^f^
Sartorius, 2011 [25]^r^			1992–2007		46,675	565	Mother died while child was 1–4 years	Child mortality at age 1–4 years	IRR = 5.17 (2.84–8.29)^g^
Finlay, 2015 [10]	Tanzania	Rural	1996–2012	Cohort	77,777	6444	Mother died when child was 0–42 days	Child mortality at ages:	
								0–30 days	RR = 6.47 (3.25–12.87)^a^
								1-6 months	RR = 20.68 (12.71–33.64)^a^
								6–12 months	RR = 2.81 (0.74–10.73)^a^
								1–2 years	RR = 6.86 (2.19–21.45)^a^
							Mother died when child was 0–365 days	Child mortality at ages:	
								0–30 days	RR = 6.42 (3.22–12.79)^a^
								1-6 months	RR = 22.48 (15.16–33.32)^a^
								6–12 months	RR = 11.60 (7.36–18.30)^a^
								1–2 years	RR = 9.79 (5.66–16.91)^a^
								2–3 years	RR = 1.66 (0.23–11.85)^a^
								3–4 years	RR = 3.79 (0.54–26.73)^a^
Katz, 2003 [30]	Nepal	Rural	1994–1997	Cohort	15.469	940	Mother died when child was 0–7 days	Child mortality at age 0–7 days	OR = 6.43 (2.35–17.56)^j^
							Mother died when child was 0–28 days	Child mortality at age 8–28 days	OR = 11.73 (3.82–36.00)
							Mother died when child was 0–24 weeks	Child mortality at age 4–24 weeks	OR = 51.68 (20.26–131.80)^j^
Masmas, 2004 [27] (rural cohort)	Guinea-Bissau	Rural	1990–1996	Cohort	633	105	Mother died when child was 0–42 days	Child mortality at age 0–12 years	MR = 3.96 (2.17–7.22)^i^
							Mother died when child aged 6 weeks-6 months	Child mortality at age 6 weeks-12 years	MR = 5.61 (2.07–15.21)^i^
							Mother died when child aged 6–12 months	Child mortality at age 6 months-12 years	MR = 4.96 (1.09–22.48)^i^
							Mother died when child aged 1–2 years	Child mortality at age 1–12 years	MR = 9.63 (2.43–38.11)^i^
							Mother died when child aged 2–5 years	Child mortality at age 2–12 years	MR = 1.64 (0.44–6.16)^i^
							Mother died when child was 0–5 years	Child mortality at age 0–12 years	MR = 4.24 (2.78–6.47)^h^
							Mother died before child aged 5 years and died 0–5 months ago	Child mortality at age 0–12 years	MR = 5.95 (3.44–10.26)^h^
							Mother died before child aged 5 years and died > 6 months ago	Child mortality at age 0–12 years	MR = 2.56 (1.29–5.09)^h^
Masmas, 2004 [27] (urban cohort)	Guinea-Bissau	Urban	1990–1997	Cohort	494	33	Mother died when child was 0–42 days	Child mortality at age 0–18 years	MR = 1.39 (0.37–5.20)^h^
							Mother died when child aged 6 weeks-6 months	Child mortality at age 0–18 years	No estimate due to skewed distribution of deaths
							Mother died when child aged 6–12 months	Child mortality at age 6 months-18 years	MR = 7.29 (0.74–71.67)^h^
							Mother died when child aged 1–2 years	Child mortality at age 1–18 years	MR = 2.01 (0.28–14.31)^h^
							Mother died when child aged 2–10 years	Child mortality at age 2–18 years	MR = 2.13 (0.60–7.56)^h^
							Mother died when child was 0–10 years	Child mortality at age 0–18 years	MR = 2.46 (1.17–5.18)^h^
							Mother died before child aged 10 years and died 0–5 months ago	Child mortality at age 0–18 years	MR = 3.09 (1.27–7.49)^h^
							Mother died before child aged 10 years and died > 6 months ago	Child mortality at age 0–18 years	MR = 1.77 (0.61–5.11)^h^
Moucheraud, 2015 [31]	Ethiopia	Rural & Town	1987–2011	Cohort	17,993	1535	Mother died when child was 0–42 days	Child mortality at ages	
								0–30 days	RR = 57.24 (25.31–129.49)^l^
								1-6 months	RR = 80.38 (21.93–294.59)^l^
							Mother died when child was 0–12 months	Child mortality at ages:	
								0–30 days	RR = 19.42 (9.24–40.85)^l^
								1-6 months	RR = 27.96 (11.11–70.39)^l^
								6–12 months	RR = 19.47 (4.85–78.18)^l^
Nakiyingi, 2003 [32]	Uganda	Rural	1989–2000	Cohort	3727	415	Mother died < 12 months ago	Child mortality at age 0–11 years	RR = 4.96 (2.35–10.47)^o^
Ng’weshemi, 2003 [33]	Tanzania	Rural	1994–2001	Cohort	6049	584	Mother died when child was 0–12 months	Child mortality at age 0–12 months	RR = 6.59 (3.51–9.50)^p^
Ronsmans, 2010 [9]	Bangladesh	Rural	1982–2005	Cohort	144,861	14,868	Mother died when child was 0–10 years	Child mortality at ages:	
								0–1 month	RR = 8.35 (5.73–12.18)^m^
								1-5 months	RR = 27.61 (20.27–37.61)^m^
								6–11 months	RR = 18.74 (11.70–30.01)^m^
								12–23 months	RR = 8.20 (5.34–12.61)^m^
								24–35 months	RR = 2.85 (1.35–6.02)^m^
								36–47 months	RR = 2.53 (1.05–6.13)^m^
								48–59 months	RR = 5.09 (2.52–10.28)^m^
								60–119 months	RR = 2.13 (1.11–4.07)^m^
Saleem, 2014 [34]	6 LMICs (Argentina, Guatemala, India, Kenya, Pakistan and Zambia)	Rural & semi urban	2010–2012	Cohort	205,737	9362	Mother died when child was 0–42 days	Child mortality at ages: 0–7 days 0–27 days	RR = 3.94 (2.74–5.65)^o^ for 0–7 days RR = 7.36 (5.54–9.77)^o^ for 0–27 days
Scott, 2017 [35]	Gambia	Rural & Urban	1989–2015	Cohort	28,502	2221	Mother died when child was 0–10 years	Child mortality at ages:	
								0–7 days	HR = 3.05 (1.12–8.28)^n^
								8–28 days	HR = 6.99 (2.98–16.36)^n^
								1–5 months	HR = 4.81 (2.30–10.06)^n^
								6–11 months	HR = 1.12 (0.23–5.35)^n^
								12–23 months	HR = 3.63 (1.56–8.47)^n^
								24–119 months	HR = 0.93 (0.37–2.33)^n^
								0–119 months	HR = 4.66 (3.15–6.89)^n^

*CI* Confidence interval, *HIV* Human immunodeficiency virus, *IRR* Incidence rate ratio, *MR* Mortality rate ratio, *NR* Not reported, *OR* Odds ratio, *RR* Rate/risk ratio, *HR* Hazard Ratio

^a^Poisson regression adjusting for child sex, twinship, mother’s age, mother’s education and household wealth

^b^Cox proportional hazards model which included factors found to be associated with childhood mortality in this population ie sex, age, year of birth, ethnic group, religion, age of mother at birth of child, season of birth, twin birth, birth order, distance from health centre, time till birth of next sibling, time since last sibling was born and vital status of last sibling

^c^Relative risk regression model which included sex, age and year

^d^Multi-level logistic regression model which included sex, age, year, multiple birth and mother’s cause of death

^e^Multi-level logistic regression model which included sex, age, year, time before and after mother’s death and multiple birth

^f^Spatio-temporal multivariate model which included year of birth, number of household deaths, previous death of sibling or stillbirth, gender, pregnancy parity

^g^Spatio-temporal multivariate model which included year, age, paternal death before age 5, number of children in household aged < 5

^h^Cox proportional hazards model corrected for age, gender, residence and orphan’s age at mother’s death

^i^Cox proportional hazards model corrected for age, gender and residence

^j^Logistic regression model which included maternal age, maternal and paternal education, sex, previous miscarriages, prior child deaths, parity, gestational age

^k^Adjusted for family and child’s gender

^l^Adjusted for household wealth, mother’s age, mother’s marital status and mother’s educational attainment

^m^Adjusted for year of birth, district, sex, gravida, maternal education and age, husband’s (SES)

^n^Adjusted for year of birth, rural/urban, mother’s age, birth spacing

^o^Univariate analyses- not adjusted for any confounders including age

^p^Effect estimate calculated from published data - not adjusted for any confounders including age

^r^Results from same population at different data collection periods

Overall, a total of 582,014 children with survival status of their biological mother were followed up and a total of 39,610 child deaths were observed. Two papers (Katz et al., 2003 & Saleem et al., 2014) followed 221,206 children aged 0–1 year and reported 10,302 child deaths [30, 34], 5 papers (Becher et al., 2004; Finlay et al., 2015; Moucheraud et al., 2015; Nakiyingi et al., 2003; Ng’weshemi et al., 2003) followed 115,668 children aged 0–5 years and reported 10,318 deaths [10, 29, 31–33], 3 papers (Houle et al.,2015; Ronsmans et al., 2010; Scott et al., 2017) followed 243,781 children aged 0–10 years and reported 18,836 deaths [9, 26, 35], 2 papers (Masmas et al., 2004 & Anderson et al., 2007) followed 865 children aged 0–12 years and reported 121 child deaths [27, 28], and 1 paper (Masmas et al., 2004) followed 494 children aged 0–18 years and reported 33 child deaths [27]. The sample sizes for each included paper ranged from 232 to 205,737 children. The time-to-event survival analysis method was used to measure the number of child deaths associated with survival status of mother. Regarding the estimation of the mortality risk, 12 of 15 articles used multivariate regression models to estimate adjusted risks of childhood mortality associated with death of a mother. The adjusted effect sizes ranged from 0.93 to 80.38, covering different stratified age ranges. While most papers reported risks of childhood mortality associated with death of mother from birth up until a specific age, three papers from two studies [24, 25, 27] reported risks of childhood mortality by multiple discrete sequential ranges of age of a child at the time of the mother’s death. Most of the included papers considered a number of potential confounders, including age, education, socioeconomic condition, parity, age at first child of mother; and gender and age when child died. Detailed study characteristics are available in Additional file 2: Table S3.

It should be noted that although we excluded studies that specifically focused on HIV positive populations, nine out of the 12 included cohorts resided in countries (South Africa, Tanzania, Burkina Faso, Guinea-Bissau, Ethiopia, Uganda, Zambia, Kenya) where the HIV prevalence among people aged 15–49 ranged from 5% to ~ 15% during 1990–2000, around the time when the studies were carried out, and during a period when antiretroviral therapy was not yet readily available [36–39].

### Risk of bias

Pre-specified potential confounders were HIV (if prevalent in cohort setting), socioeconomic status and age of child for the purpose of assessing the comparability of exposed and non-exposed cohorts. The risk of bias was assessed as either low or medium for each of the 12 items assessed for 9 of the 16 sets of data (four sets of data were reported for one of the 13 cohorts). Six reports were assessed at high risk of bias due to confounding, one was assessed at high risk of bias due to differences in follow-up between exposed and non-exposed, and one was assessed at high risk of bias due to incompleteness of follow-up. Full details can be found in Table 2 and Additional file 2: Table S4.

**Table 2:** Risk of bias of the 16 data sets of 13 cohorts reporting in 15 articles of 12 original studies

	Scott, 2017 [35]	Finlay 2015 [10]	Moucheraud, 2015 [31]	Houle, 2015 [26]	Clark, 2013 [11]	Sartorius, 2011 [25]	Saleem, 2014 [34]	Ronsmans, 2010 [9]	Sartorius, 2010 [24]	Anderson, 2007 [28]	Becher H, 2004 [29]	Masmas, 2004 (Rural) [27]	Masmas, 2004 (Urban) [27]	Nakiyingi, 2003 [32]	Ng’weshemi, 2003 [33]	Katz J, 2003 [30]
Bias in selection of participants into study	Low	Low	Low	Low	Low	Low	Low	Low	Low	Low	Low	Low	Low	Low	Low	Low
Measurement of exposure^a^	Moderate	Moderate	Moderate	Moderate	Moderate	Moderate	Moderate	Moderate	Moderate	Moderate	Moderate	Moderate	Moderate	Moderate	Moderate	Moderate
Measurement of outcome	Moderate	Moderate	Moderate	Moderate	Moderate	Moderate	Low	Moderate	Moderate	Moderate	Moderate	Moderate	Moderate	Moderate	Moderate	Moderate
Was outcome of interest absent at the time to which the exposure refers?	Moderate	Moderate	Moderate	Moderate	Moderate	Moderate	Moderate	Moderate	Moderate	Moderate	Moderate	Low	Low	Moderate	Moderate	Moderate
Was follow-up long enough for outcome to occur as a consequence of measured exposure?^b^	Low	Low	Low	Low	Low	Low	Low	Low	Low	Low	Low	Low	Low	Low	Low	Low
Participation rate	Low	Low	Low	Low	Low	Low	Low	Low	Low	Low	Low	Low	Low	Low	Low	Low
Completeness of follow-up	Low	Low	Low	Low	Low	Low	Low	Low	Low	High	Moderate	Low	Moderate	Low	Moderate	Low
Accuracy of dates of outcomes or censoring	Low	Low	Low	Low	Low	Low	Low	Low	Low	Low	Low	Low	Low	Low	Low	Low
Difference in follow-up between exposed and non-exposed	Low	Low	Low	Low	Low	Low	Low	Low	Low	Low	Low	Low	High	Low	Low	Low
Difference in missing data for exposure between those with or without the outcome^c^	Low	Low	Low	Low	Low	Low	Low	Low	Low	Low	Low	Low	Low	Low	Low	Low
Comparability of exposed and non-exposed cohorts with respect to potentially important confounding variables^d^	High	Low	Moderate	Low	Moderate	Moderate	High	Low	High	High	High	Low	Low	High	Moderate	Low
Covariates are appropriately included in statistical analysis models	Low	Low	Low	Low	Low	Low	Low	Low	Low	Low	Low	Low	Low	Low	Low	low

^a^Rated at low risk of bias when pre-existing data sets with exposures and outcomes reported by different or same databases or and authors state outcomes determined blind to exposure; Rated at moderate risk if exposure measured by looking at pre-existing records containing both exposures and outcomes and no mention of blinding or using a structured interview

^b^Adequate follow-up could be any time for children less than 2 years old, but at least 1 year for children > 2 years

^c^Rated as low risk if data were from Census and Health and Demographic Surveillance System (HDSS) data

^d^Potentially important prespecified confounders were age of child, HIV infection if prevalent in cohort setting and SES/Education

### Estimated effects of child mortality according to maternal vital status

#### Pooled relative risks of childhood mortality by age of child when mother died

**Fig. 2:**
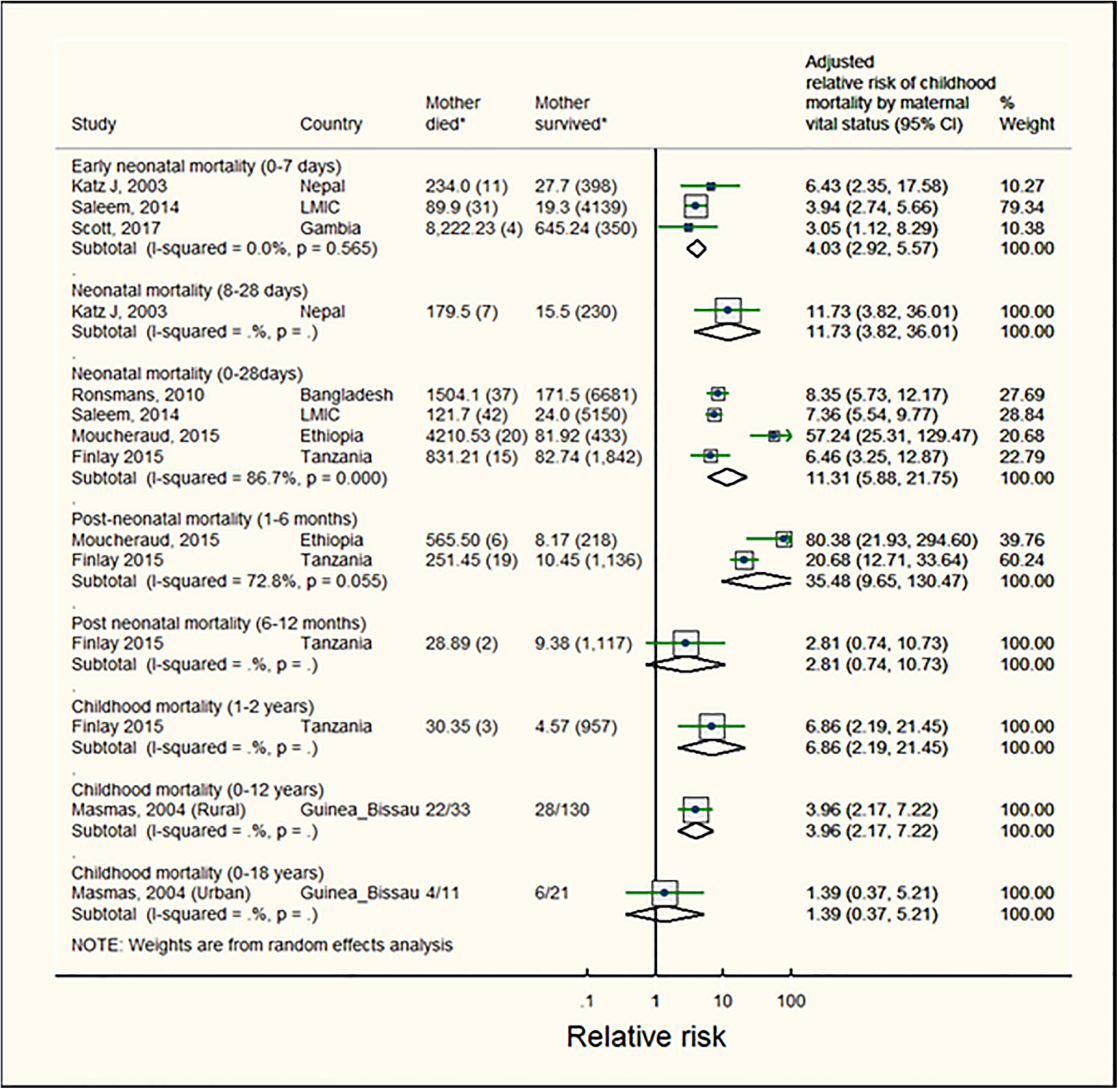
Mortality risks for children whose mother died when child was aged up to 42 days. Note: The sizes of boxes on study-specific 95% confidence intervals are proportional to % weight. Diamond symbols represent the 95% confidence interval for the pooled estimate of each sub-group. (*)Numbers represent mortality rate (number of child deaths) or child deaths/total children in mother died (exposed) and mother survived (unexposed) groups. These numbers were extracted from the studies but not directly use to estimate effect sizes. Effect estimates were extracted from the study reports and were usually estimated from multivariate regression models. Katz et al. [30] and Saleem et al. [34] reported deaths per 1000 live births (n deaths). Scott et al. [35] reported mortality rate per 1000 child years (n deaths). Ronsmans et al. [9], Finlay et al. [10], Moucheraud et al. [31] reported deaths per 100,000 child-days (n deaths). Masmas et al. [27] reported number of child deaths per total number of children

For children whose mother died when they were ≤ 0–42 days, the RR of dying at 0–28 days was 11.3 (95%CI: 5.9–21.8, p [het] = 0.001, pooled estimate from 4 studies), at 1–6 months this risk increased to 35.5 (95%CI:9.7–130.5, p [het] = 0.05, pooled estimate from 2 studies), compared to those whose mothers did not die; at 6–12 months the RR substantially dropped to 2.8(95%CI: 0.74–10.73, one estimate). The effect of mother’s death on child mortality was markedly lower after children reached 1 year of age (Fig. 2 and Table 3a).

**Table 3:** Risk estimates for childhood mortality, stratified by exposure and outcome

a.) Pooled risk of child mortality stratified by death of a mother when child aged 0–42 days, 0–12 months, 0–5 years and 0–10 years														
Age of child at mother’s death (exposure)	Age at risk of child death (outcome)													
	0–7 days	8–28 days	0–28 days	1–6 months	6–12 months	0–1 years	1–2 years	2–3 years	3–4 years	4–5 years	5–10	0–10	0–12 years	0–18 years
0–42 days^a^	4.03 (2.92–5.57)^a^	11.73 (3.82–36.01)	11.31 (5.88–21.75)^b^	35.48 (9.65–130.47)^c^	2.81 (0.74–10.73)		6.86 (2.19–21.45)						3.96 (2.17–7.22)	1.39 (0.37–5.21)
0–12 months^m^			11.06 (3.74–32.74)^d^	27.45 (17.61–42.81)^e^	12.20 (7.91–18.81)^f^	15.90 (2.1–16.10)^g^	9.79 (5.66–16.91)	1.66 (0.2–11.86)	3.79 (0.54–26.72)					
0–5 years^n^													4.12 (2.96–5.74)^h^	
0–10 years^o^		6.99 (2.98–16.38)		11.92 (2.15–65.96)^i^	5.08 (0.32–79.80)^j^		5.94 (2.72–12.97)^k^	2.85 (1.30–6.02)	2.53 (1.05–6.11)	5.09 (2.52–10.28)	2.13 (1.11–4.08)	4.66 (3.1–4.89)		3.67 (2.01–6.73)
b) Relative risk of childhood mortality stratified by mother’s death at multiple discrete sequential intervals of child age														
Included articles			Number of studies, n exposed/unexposed deaths			Age of child at mother’s death (exposure)			Age of child at child death			Risk estimate (95% CI) (Outcome)		
Masmas, et al., 2004 (rural and urban cohorts) [27]			2 cohorts, 26/34 child deaths			0–42 days			Birth up to 18 years			2.79 (1.06–7.34), meta-analysis, p [het] = 0.28^p^		
			1 cohort, 10/12 child deaths			42 days- < 6 months			6 weeks-12 years			5.61 (2.07–15.21)		
			2 cohorts from one study, 7/7 child deaths			6–11 months			6 months up to18 years			5.58 (1.58–19.70), meta-analysis, p [het] = 0.78)^p^		
			2 studies (3 cohorts), 9 child deaths			1- < 2 years			1 year up to 18 years			5.18 (1.16–23.26), meta-analysis, p [het] = 0.20)^p^		
			2 cohorts from one study, 9 child deaths			≥2 years			2 years up to18 years			1.88 (0.75–4.69), meta-analysis, p [het] = 0.78)^p^		
Sartorius et al., 2010 and 2011 [24, 25]			1 cohort, 91child deaths (30% of mother died of HIV/TB)			0–1 year			0–1 year			51.11 (8.49–200.80)		
			1 cohort, 191 child deaths (not due to HIV/TB)			1–4 years			1–4 years			5.17 ((2.84–8.29)		
c) Risks of child mortality by time since mother’s death														
			Number of studies, n exposed/n unexposed deaths			Time since mother’s death (exposure)			Age at risk of child’s death			Relative risk of child death, 95%CI		
Clark et al., 2013 [11]			1 estimate, 193/1,537,831 observed child-months			0 month			0–59 months			12.55 (6.22–25.34)		
Clark et al., 2013 [11]			1 estimate, 295/1,537,831observed child-months			1–2 months			0–59 months			7.01 (3.16–15.56)		
Clark et al., 2013 [11]			1 estimate, 740/1,537,831observed child-months			3–5 months			0–59 months			4.03 (1.53–10.58)		
Masmas, et al., 2004 (rural and urban cohorts) [27]			2 estimates, 46/74 child deaths			0–5 months ago			0 up to 18 years			4.72 (2.56–8.72), meta-analysis, p [het] = 0.22^q^		
Masmas, et al., 2004 (rural and urban cohorts) and Clark et al., 2013 [11, 27]			3 estimates, 18/74 child deaths			> 6 months ago			0 up to 18 years			2.08 (1.27–3.41), meta-analysis, p [het] = 0.69^q^		

**Fig. 3:**
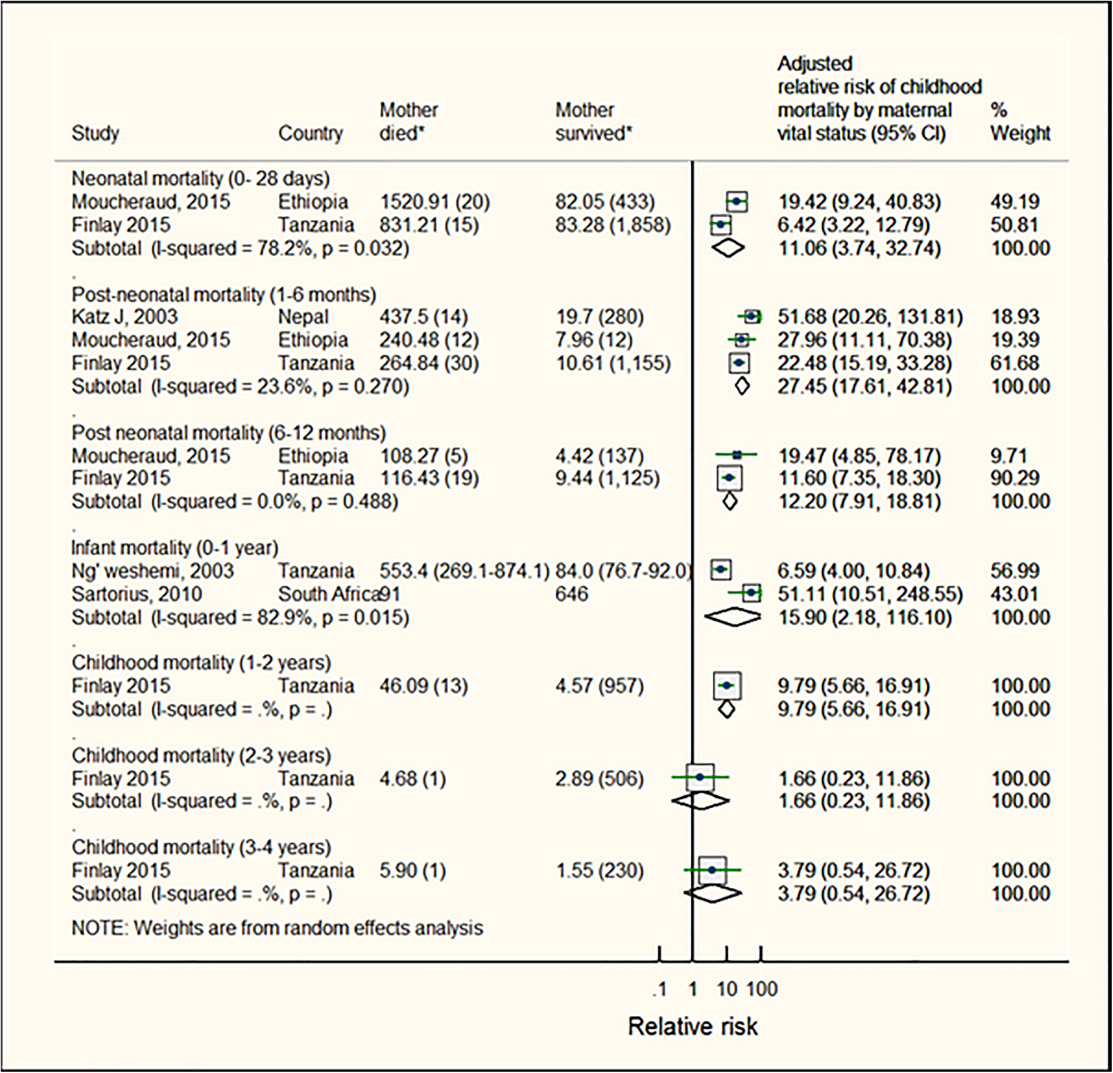
Mortality risks for children whose mother died when child was aged up to 12 months. Note: The sizes of boxes on study-specific 95% confidence intervals are proportional to % weight. Diamond symbols represent the 95% confidence interval for the pooled estimate of each sub-group. (*) Numbers represent mortality rate (number of child deaths) or child deaths/total children in mother died (exposed) and mother survived (unexposed) groups. These numbers were extracted from the studies but not directly use to estimate effect sizes. Effect estimates were extracted from the study reports and were usually estimated from multivariate regression models. Katz et al. [30] and Ng’weshemi et al. [33] reported deaths per 1000 live births (n deaths). Scott et al. [35] reported mortality rate per 1000 child years (n deaths); Sartorius et al. [24] reported incident rate ratios. Finlay et al. [10] and Moucheraud et al. [31] reported deaths per 100,000 child-days (n deaths)

**Fig. 4:**
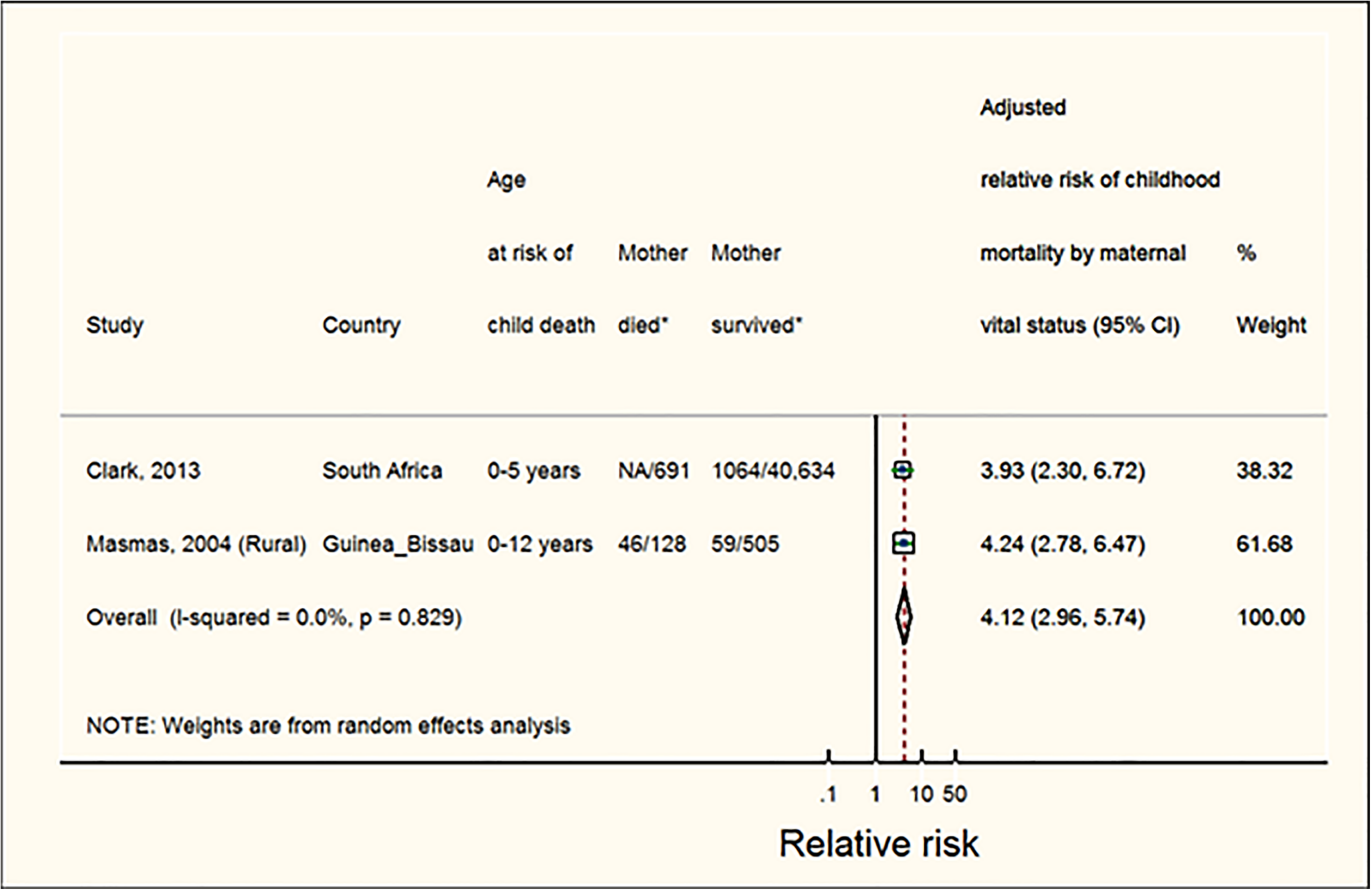
Mortality risks for children whose mother died when child was aged 0–5 years. Note: The sizes of boxes on study-specific 95% confidence intervals are proportional to % weight. Diamond symbols represent the 95% confidence interval for the pooled estimate of each sub-group. (*) Number represent child deaths/total children in mother died (exposed) and mother survived (unexposed) groups. These numbers were extracted from the studies but not use to estimate effect sizes. Effect estimates were extracted from the study reports and were usually estimated from multivariate regression models. Clark et al. [11] reported number of all causes child deaths (HIV/TB-related deaths were excluded)/ total number of children; Masmas et al. [27] reported number of child deaths/ total number of children

Pooled estimates: ^a^pooled estimate based on three studies, exposed deaths:46; ^b^pooled estimate from four studies, exposed deaths = 114; ^c^pooled estimate from two studies, exposed deaths = 25; ^d^pooled estimate from two studies, exposed deaths = 35; ^e^pooled estimate from three studies, exposed deaths = 56; ^f^pooled estimate from two studies, exposed deaths = 24; ^g^pooled estimate from two studies, exposed deaths = NR (Ng’weshemi et al. [33] did not reported number of child deaths for 0–1 year); ^h^pooled estimate from two studies, exposed deaths = 124; ^i^pooled estimate from two studies, exposed deaths = 57; ^j^ pooled estimate from two studies, exposed deaths = 22; ^k^pooled estimate from two studies, exposed deaths = 26; remaining estimates were from one study only; ^l^see Fig. 1; ^m^see Fig. 2; ^n^see Fig. 3; ^o^see Fig. 4; ^p^see Additional file 3: Figure S7A; ^q^see Additional file 3: Figure S7B;

A high level of heterogeneity (I^2^ = 86.7% (95%CI: 68–94%, p [het] = 0·001) was observed between the four individual neonatal mortality estimates (0–28 days), primarily due to higher estimate of Moucheraud et al. [31] (in general, the effect estimates by Moucheraud et al. [31] were higher than those reported from other studies). A sensitivity analysis excluding this study from the neonatal mortality (0–28 days) strata reduced the pooled estimate from 11.3 to 7.6 (95%CI: 6.1–9.4; p [het] = 0.78) (Additional file 3: Figure S8B).

For children whose mother died when they were ≤ 0–12 months, the RR of dying before 12 months of age was 15.9 (95%CI: 2.2–116.1; p [het] = 0.02, pooled estimate from 2 studies,) (Fig. 3 and Table 3a). The stratified RRs of dying before 1 month, 1–6 months and 6–12 months of age were 11.1 (95%CI: 3.7–32.7; p [het] = 0.03, pooled estimate from 2 studies); 27.5 (95%CI: 17.6–42.8; I^2^ = 23.6% (95%CI: 0–92%) p [het] = 0.3, pooled estimate from 3 studies); and 12.2 (95%CI: 7.9–18.8, p [het] = 0.5, pooled estimate from 2 studies), respectively. For children aged 2–3 years and 3–4 years the estimated relative risks from single studies were not significant.

Overall, children whose mother died before they turned 5 years had a pooled RR of death up to 12 years at 4.1 (95%CI: 3.0–5.7; p [het] = 0.83). Results were based on 2 estimates from 2 studies; (Fig. 4 and Table 3a).

**Fig. 5:**
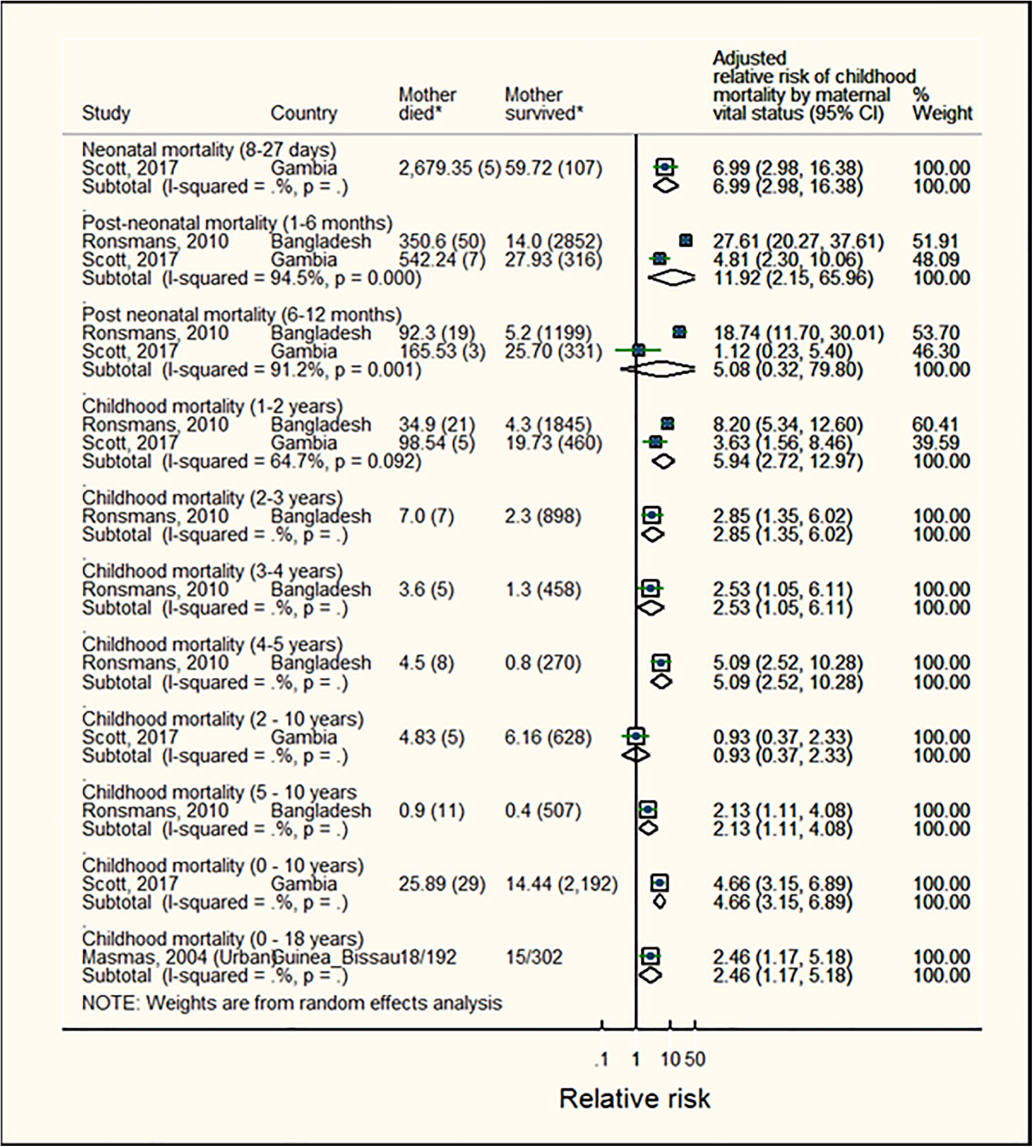
Mortality risks for children whose mother died when child was aged up to 18 years. Note: The sizes of boxes on study-specific 95% confidence intervals are proportional to % weight. Diamond symbols represent the 95% confidence interval for the pooled estimate of each sub-group. (*)Numbers represent mortality rate (number of child deaths) or child deaths/total children in mother died (exposed) and mother survived (unexposed) groups. These numbers were extracted from the studies but not directly use to estimate effect sizes. Effect estimates were extracted from the study reports and were usually estimated from multivariate regression models. Scott et al. [35] reported mortality rate per 1000 child years (n deaths); Ronsmans et al. [9] reported deaths per 100,000 child-days (n deaths); Masmas et al. [27] reported number of child deaths/ total number of children

For children who were ≤ 10 years and up to 18 years of age when their mother died, the RR of dying before 10 years was 4·7 (95%CI: 3.2–6.9; result was based on one study) and before 18 years was 2.5 (95%CI: 1.2–5.2; result was based on one study) (Fig. 5 and Table 3a). Stratified analyses by subgroups of children whose mother died any time ≤ 10 years of age showed that the RR of childhood mortality was highest among children who died at 1–6 months (RR = 11.9 (95%CI: 2.2–66.0), p [het] = 0.0001, 2 estimates) and reduced for older children (RR = 5.1 (95%CI: 0.3–79.8); 5.9 (95%CI: 2.7–13.0); 2.9 (95%CI: 1.4–6.0) among children aged 6–12 months, 1–2 years, and 2–3 years, respectively). Only one study reported the risk of childhood mortality at 5–10 years of age with RR = 2.13 (95%CI: 1.11–4.08).

#### Mortality risks for children whose mother died at multiple discrete sequential intervals of child’s age

Apart from risks of child death categorized by the four main groups of child’s age at death of a mother as reported above, other exposure groups of mother’s death stratified by discrete sequential age intervals at 0–42 days, 42 days- < 6 months, 6–11 months, 1- < 2 years, ≥2 years, 0–1 year, and 1–4 years were also reported in three papers (Masmas et al. [27] (two cohorts) and Sartorius et al. [24, 25]). Results from these primary studies indicated that the mortality risk for children whose mother died at different discrete intervals of child age (as mentioned above) were consistent with the trend towards lower relative risks with increasing age at time of mother’s death. The Masmas et al. study indicated that children whose mother died at 0–42 days, 42 days- < 6 months, 6–11 months and 1- < 2 years of their life had higher relative risks of childhood mortality, compared to those whose mother lived (RR = 2.8 [95%CI: 1.1–7.3]; 5.6 [2.1–15.2]; 5.6 [1.6–19.7]; and 5.3 [3.3–8.6], respectively) than those whose mother died when the child was older (≥2 years) (RR = 1.9 [95%CI: 0.8–4.7]). The results from the study by Sartorius et al. were consistent with these findings, although the risk of child mortality in children who experienced the death of a mother was substantially higher, an effect which is possibly relating to the higher prevalence of HIV infection in South Africa. These studies also confirmed that there was an increased risk of child death due to a mother’s death, even when the mother died when the child was older than 42 days (42 days- < 6 months, 6–11 months and even ≥2 years). The results for this sub-group analysis are presented in Table 3b and detailed forest plots of meta-analyses for pooled estimates as shown in Table 3b are presented in Additional file 3: Figure S7A.

#### Childhood mortality risk by ‘time since mother’s death’

The relative risk of childhood mortality in relation to ‘time since mother’s death’ was reported for 3 cohorts. Among children whose mothers died, the risk of mortality was 4.7 times higher (95%CI: 2.6–8.7; p [het] = 0.2; pooled estimate from 2 cohorts) during the 6 months following a mother’s death and 2.1 times higher (95%CI: 1.3–3.4; p [het] = 0.7; pooled estimate from 3 cohorts) after 6 months following a mother’s death, compared to the overall mortality among children whose mother survived (Table 3c and Additional file 3: Figure S7B).

#### Results of sensitivity analysis

For children whose mother died before 42 days of age, a high level of heterogeneity was observed between the four studies included in the neonatal mortality strata (0–28 days). Results of sensitivity analysis suggested that the high level of heterogeneity which was observed in the pooled estimate of child mortality of children aged 0–28 days was probably not related to country HIV status but due to an outlier (estimate of Moucheraud et al. [31]) (Additional file 3: Figure S8A and S8B).

Sensitivity analyses stratifying for OR risk estimates were conducted to identify sources of heterogeneity of pooled estimates of child mortality for children whose mother died when the child was aged 0–42 days and pooled estimates of child mortality at age 1–6 months for children whose mother died when the child was aged 0–1 year. The results of sensitivity analyses stratifying OR vs. Rate Ratio/Risk Ratio/Hazard Ratio showed insignificant differences for both cases (heterogeneity still exists after stratifying for OR) (Additional file 3: Figure S8C and S8D).

## Discussion

Our review of 15 articles reporting 13 cohorts of 12 original studies included in qualitative analysis, of which 13 articles reporting 11 cohorts of 11 original studies included in meta-analysis, showed that the relative risks of childhood mortality varied by the age of the child at the time of the mother’s death, time since the mother died, and age-at-risk of child death. Overall, we found strong evidence of an association between death of a mother and childhood mortality in lower resource settings. Compared to overall mortality among children whose mother survived, the risk of mortality associated with a mother’s death appeared to be highest for children in their first 6 months of life (~ 35 times) and this risk decreased (to ~ 28 times) for children up to 1 year of age at the time of a mother’s death. Results of sub-group analyses at different discrete sequential intervals of child age suggested that there was an increased risk of childhood mortality for children up to 18 years among children whose mother died when the child was older than 42 days after birth. We also found that the risk of death was higher in the first 6 months following a mother’s death (~ 4.7 times) than it was after 6 months (~ 2.1 times).

The high risk of mortality among children whose mother died in their first 6 months of age is understandable. Reduced breastfeeding which can cause malnutrition and increased risk of death in neonates and obstetric complications at delivery, such as haemorrhage (which is a leading cause of pregnancy-related maternal mortality and subsequent neonate mortality due to asphyxia) are likely to be major contributors to the higher risk of mortality [40–42]. The risk remained high among children aged 1–2 years whose mother died at any age of the child. It is possible that lack of breast feeding, diarrhoea, respiratory infections, and nutrition disorders might increase the risk of childhood mortality at these ages [9, 31]. As Ronsmans et al., 2010 and Moucheraud et al., 2015 discussed, in settings where up to 90% of infants were breast fed, the risk of child death following a death of a mother was substantial [9, 31]. The risk also appeared elevated for children aged 5–10 years and up to 18 years. It is possible that lack of mother’s care in daily life, particularly when children are ill might be associated with risk of childhood mortality at older ages [9]. Child death up to 12 or 18 years of age, following a mother’s death, might be associated with other factors, including disease, malnutrition, poor hygiene, injuries, and psychological/mental problems [43, 44]. However, mortality risks for adolescents were reported by three studies only and effects among children who were followed up until aged 18 years could be predominantly driven by child mortality at a younger age. A previous review found that the mortality risk of children under 5 years who lost their mother was more than 4 times higher than the risk of those whose mother survived [12]; our findings are generally consistent with the previous results. However, our review provides more insight into the association between mother and child mortality in LMICs through a detailed analysis which stratified the results by both outcomes (childhood mortality) and exposures (mother’s death).

Our findings emphasise the critical importance of women in familial outcomes and the importance of health care for women through their child rearing years in lower resource countries. As almost all included studies were conducted in rural and semi-urban areas of low-income countries, lower-middle income countries, and upper-middle-income countries, the findings cannot be directly interpreted in the context of high income settings. Our findings suggested that the relative risk of childhood mortality following a death of a mother was substantially higher for younger children (0–1 year) than for older children (≥ 2–18 years). This can be explained by the numerous factors that affect the stages of child development. For children less than 1 year of age, losing a mother will potentially expose the child to malnutrition and infectious diseases due to lack of breastfeeding and maternal care [45, 46]. Ronsmans found that among motherless children who died in their first year, 50% died of infectious diseases (diarrhoea and respiratory infections) and 16% died from malnutrition [9]. As Moucheraud et al., 2015 concluded, in a setting where breastfeeding is a sole source of nutrition for newborns and infants and substitute food sources are unaffordable, inaccessible or unsafe, the risk of child death following a death of a mother will be elevated [31]. For older children, lack of maternal care during daily life and when ill is likely to drive the increased risk of death, especially in settings where there are no extended family or social support structures for motherless children [8]. It has been shown that in settings where motherless children can receive care from grandmothers or adopted families, the survival rate of those children was significantly higher than motherless children without such care givers [47, 48].

This systematic review and meta-analysis has several strengths. We reviewed and synthesized in detail the evidence published on the impact of death of a mother on childhood mortality in lower-resource settings. The review was independently conducted by two reviewers, and we followed best practice in systematic review methodology. The majority (11/12) of included studies were cohort studies, one of the best designs for risk factor studies [49]. Overall, settings were relatively homogenous among studies, with most conducted in rural or semi-urban areas of lower resource settings. Additionally, to account for the impact of HIV on child mortality, we only included studies that were conducted in the general population, and sensitivity analyses were used to check for the impact of HIV prevalence of the study settings on the pooled estimates. Furthermore, given each of the included papers measured death of a mother and child mortality in various outcome and exposure categories, pooled analyses stratified by these categories were conducted to account for heterogeneity. However, given a number of studies reported several mortality risks at different child ages (or a number of original studies were associated with multiple reports), we did not pool the relative risk if more than one estimate was reported from an original study. Additionally, we conducted careful risk of bias assessment for all included reports. Our assessments found that 57% (9/16) data sets of 15 included reports in the risk of bias assessment had low or medium risk of bias for 12 assessed items. Potential confounders for both death of a mother and childhood mortality, including age of child, family socioeconomic status, and population after and before HIV status, were carefully evaluated when assessing for the risk of bias of each individual report. Regarding the qualities of the extracted data, given all included studies used various methods of survival analysis models to produce the relative risks of childhood mortality according to a death of a mothers, 12 out of 15 reports generated effects sizes from multivariate regression models, whereas 2 reports [32, 33] generated effects sizes using univariate models. Finally, based on our understanding that the association between death of a mother and childhood mortality would vary by level of country development, our current review only focused on investigating the risk of death of a mother on childhood mortality in lower resource countries.

Our review has some limitations. First, at the study level, given our search was limited in English language, we potentially missed a number of studies in other languages. As a result the number of studies that met the eligible criteria comprised 12 original studies (reported in 15 papers). Second, although study designs, study settings, and time periods over which data were collected were similar among the included studies, there was unexplained variation among studies for some strata. Possible sources of heterogeneity include the differences among country-specific study settings in socioeconomic conditions, culture, disease patterns, and health care systems. Regarding the methodology of the original studies, there was variation in sample sizes, quality and design of the study (different length of follow-up, level of detail reported), and methods used to measure the mortality rates (e.g. some analyses reported rates per 1000 live-births, others reported mortality rate per 1000 child-year or per 100,000 child-days). Given these diversities exist, heterogeneity in pooled analyses is understandable. We presented pooled estimates from meta-analysis even in the presence of significant heterogeneity because it has been argued that such analyses can still be appropriate and useful provided effect estimates from individual studies are in the same direction and random effect methods are used [50, 51]. Third, based on the available data extracted from included studies, our meta-analyses were conducted using variously reported effect estimates (mortality rate ratio, risk ratio, odd ratio and hazard ratio). However, sensitivity analysis stratifying OR vs. Rate Ratio/Risk Ratio/Hazard Ratio showed insignificant differences. Additionally, for many of the age intervals, pooled estimates were based on only one or two studies. Consequently, readers should be suitably cautious about interpretation and generalisation of such estimates, carefully taking into account the levels of uncertainty implied by confidence intervals. We anticipate that HIV is likely to be confounder of the association between maternal and child mortality. Although at study selection and data extraction stages, studies assessing only mothers with HIV were excluded, we were unable to account for individual mothers with HIV in the included studies. For any pooled estimates with high heterogeneity, sensitivity analysis stratifying for HIV status suggested that there was probably not any association with country HIV status. Another limitation of this review was that most of the 12 included studies (10/ 12) reported the risks of childhood mortality among children whose mother died at any time during their childhood (0–1 year, 0–5 years, 0–18 years), probably due to limited numbers. This approach allows us to see the general impact of the death of a mother on childhood mortality. Although the magnitude of the impact could be seen up to many years later in childhood, it could be argued that most of the mother and child deaths might have been driven by deaths occurring at child birth or postpartum. There were 2 studies reported in 3 articles which analysed risks of child death by multiple discrete sequential ranges of age of a child at the time of the mother’s death. This allows for more precise measuring of the risks of childhood mortality associated with death of a mother occurring at different child ages. Results of sub-analysis based on 2 studies stratified the risks of childhood mortality by multiple discrete sequential ranges of age of a child at the time of the mother’s death were broadly consistent with the main findings and suggested that there was an effect of a mother’s death on child mortality even when the child was older than 42 days after birth. Finally, the impact of death of a mother on childhood mortality is only one of several consequences that we selected to address in the scope of this review. Psychological development, school achievement and risk behaviours of motherless children are outcomes of importance for future research [52–55].

Findings from this review emphasized the importance of the Millennium Development Goals (1990–2015) and Sustainable Development Goals (2016–2030) to reduce maternal mortality as well as childhood mortality globally, since avoiding a death of a mother has immense effects on child mortality. Skilled birth attendants and emergency obstetric care for childbirth complications have been recommended [56]. For other causes of female deaths, including HIV/AIDS, cancer, cardiovascular diseases, and other chronic diseases, targeted-disease prevention and control programs (many for both males and females) have been recommended under several initiatives. In 2011, at the World Economic Forum, WHO introduced “best buy” interventions which target lifestyle risk factors and disease, including tobacco use, harmful alcohol use, unhealthy diet and physical inactivity, and cancer [57]. Recently, new Disease Control Priorities, 3rd edition (DCP3) were published by the World Bank, and these aim to assist country decision makers in prioritizing various health issues within limited resources and budgets [58]. Many of these recommendations are related to the leading causes of female deaths in lower resource countries [58]. Our findings demonstrate that these programs will not only benefit women, but also provide benefits in terms of familial outcomes.

## Conclusions

We found evidence of an association between death of a mother and childhood mortality in lower resource settings. These findings emphasize the critical importance of women in family outcomes, and the importance of health care for women during the intrapartum and postpartum periods and throughout their child rearing years in these settings.

## Additional files

Additional file 1: Inclusion and exclusion criteria and search strategy. (DOCX 34 kb)

Additional file 2: Supportive information on detailed study characteristics, risk of bias assessment, and extracted results of studies included in meta-analysis. (DOCX 66 kb)

Additional file 3: Supportive information on additional analyses and sensitivity analysis. (DOCX 5610 kb)

Additional file 4: The Checklist of the paper that follow PRISMA. (DOC 65 kb)

## Data Availability

All data generated by this review are included in this published article and its Additional files. The included articles which were analysed in this review are accessible via existing journals.

## Abbreviations

AIDS: Acquired Immunodeficiency Syndrome
DCP3: Disease Control Priorities-3
HIV: Human Immunodeficiency Virus
HR: Hazard Ratio
IRR: Incidence Rate Ratio
LMICs: Low-and-middle-income countries
RR: Relative risk
TB: Tuberculosis
WHO: World health organization
