# Supplementary material for: Risk of childhood mortality associated with death of a mother in low-and-middle-income countries: a systematic review and meta-analysis
Source: BMC Public Health. 2019 Oct 11;19:1281. doi: 10.1186/s12889-019-7316-x (PMC6788023; doi:10.1186/s12889-019-7316-x)
Supplement: Supplementary file 1 — Inclusion and exclusion criteria and search strategy. (DOCX 34 kb) [file 12889_2019_7316_MOESM1_ESM.docx]

## Additional file 1: Supplementary information

## Table S1A : Inclusion and exclusion criteria

| **Selection criteria** | **Inclusion criteria** | **Exclusion criteria** |
| --- | --- | --- |
| **Study type** | Risk factor |  |
| **Study design** | Cohort or nested case-control studies | Cross-sectional studies |
| **Population of interest** | Children aged 0-18 years living in low, lower-middle, and upper-middle income countries as defined by World Bank | Includes individuals aged older than 18 years  Population of children in settings other than low, lower-middle, and upper-middle income countries as defined by World Bank |
| **Study factor (exposure factor)** | Death of mother by any cause e.g. maternal death, diseases, injuries or natural disasters) from birth of child until 18 years | Death of mother after child reaches 18 years of age or older  Death of parents (no analyses for maternal death)  Mother death due to HIV/AIDS  Studies restricted to a single cause of death eg pregnancy-related deaths  Mother-child separations eg. divorce or immigration  Death of father |
| **Comparators** | Mother alive at ages defining the exposure | Mother separated from child for reasons other than death eg separations due to divorce or migration |
| **Outcomes** | Report comparative risk of overall or age-specific mortality for children under 18 years according to mother’s vital status. | Report only: overall or cause-specific child mortality rate, probability of child survival, or child morbidity |
| **Data collection and publication periods** | All studies with data collection after 1979 published from 1/1/1980 to 31/3/2017 | All studies with data collected before 1980 |

## Table S1B: List of excluded papers

|  | **Excluded papers** | **Reasons of exclusion** |
| --- | --- | --- |
| 1 | Chen et al., 2015[1] | Not relevant setting (developed country) |
| 2 | Li et al., 2014[2] | Not relevant setting (developed country) |
| 3 | Pavard et al., 2005[3] | Data collected before 1979 |
| 4 | Sear et al., 2002[4] | Data collected before 1979 |
| 5 | Koenig et al., 1988[5] | Data collected before 1979 |
| 6 | Zaba et al., 2005[6] | Not relevant study population (HIV(+) mother) |
| 7 | Crampin et al., 2003[7] | Not relevant study population (HIV(+) mother) |
| 8 | Newell et al., 2004[8] | Not relevant study population (HIV(+) mother) |
| 9 | Taha et al., 1996[9] | Not relevant study population (HIV(+) mother) |
| 10 | Kazembe et al., 2012[11] | Not relevant study design (modelling) |
| 11 | Pavard et al., 2007[12] | Not relevant study design (modelling) |
| 12 | Razzaque, 2013[13] | Not relevant comparators |
| 13 | Felisbino-Mendes et al., 2015[14] | Not relevant exposure (No maternal death) |
| 14 | Diallo et al., 2012[15] | Not relevant exposure (No maternal death) |
| 15 | Taha et al., 1995[16] | Not relevant exposure (No maternal death) |
| 16 | Ezeh et al., 2015[17] | Not relevant exposure (No maternal death) |
| 17 | Binka et al., 1995[18] | Not relevant exposure (No maternal death) |
| 18 | Abir et al., 2014[19] | Not relevant exposure (No maternal death) |
| 19 | Filippi et al., 2010[20] | Not relevant exposure (No maternal death) |
| 20 | Tollman et al., 2008[21] | Not relevant exposure (No maternal death) |
| 21 | Clark et al., 2013[22] | Not relevant exposure (No maternal death) |
| 22 | Owais et al., 2013[23] | Not relevant exposure (No maternal death) |
| 23 | Zhou et al., 2016[24] | No effect estimated reported |
| 24 | Hammer et al., 2006[25] | No effect estimate reported |

## Table S2: Search strategy

Database(s): **Embase Classic+Embase**1947 to 2016 August 29**, Global Health**1910 to 2016 Week 33**, Ovid MEDLINE(R) In-Process & Other Non-Indexed Citations and Ovid MEDLINE(R)**1946 to Present**, Ovid MEDLINE(R) Daily Update**August 29, 2016 
Search Strategy:

| **#** | **Searches** | **Results** |
| --- | --- | --- |
| 1 | maternal death*.mp. | 15783 |
| 2 | maternal mortalit*.mp. | 40857 |
| 3 | maternal loss*.mp. | 207 |
| 4 | (loss* adj5 mother).mp. | 689 |
| 5 | (loss* adj5 parent).mp. | 1063 |
| 6 | parent* death*.mp. | 1027 |
| 7 | parent* mortalit*.mp. | 96 |
| 8 | (death* adj5 parent*).mp. | 4687 |
| 9 | (death* adj5 mother).mp. | 1335 |
| 10 | parent* loss*.mp. | 785 |
| 11 | parental deprivation/ | 1337 |
| 12 | parental death/ | 1244 |
| 13 | parental absence/ or exp father absence/ or exp mother absence/ | 189 |
| 14 | 1 or 2 or 3 or 4 or 5 or 6 or 7 or 8 or 9 or 10 or 11 or 12 or 13 | 54755 |
| 15 | child*.mp. | 4946713 |
| 16 | adolescen*.mp. | 3335933 |
| 17 | exp adolescent/ | 3198716 |
| 18 | youth*.mp. | 140579 |
| 19 | juvenile*.mp. | 211327 |
| 20 | child/ | 3101648 |
| 21 | adolescent/ | 3198334 |
| 22 | age*.mp. | 19462682 |
| 23 | juvenile/ | 33553 |
| 24 | 15 or 16 or 17 or 18 or 19 or 20 or 21 or 22 or 23 | 21681738 |
| 25 | (death* adj5 child).mp. | 31320 |
| 26 | (death* adj5 children).mp. | 21451 |
| 27 | juvenile mortalit*.mp. | 379 |
| 28 | child mortalit*.mp. | 10042 |
| 29 | child mortality/ | 11915 |
| 30 | (juvenile adj3 death).mp. | 225 |
| 31 | children mortalit*.mp. | 371 |
| 32 | (mortalit* adj3 child).mp. | 11682 |
| 33 | (mortalit* adj3 children).mp. | 13383 |
| 34 | 25 or 26 or 27 or 28 or 29 or 30 or 31 or 32 or 33 | 75742 |
| 35 | 14 and 24 | 33566 |
| 36 | 34 and 35 | 3873 |
| 37 | limit 36 to english language | 3409 |
| 38 | limit 37 to humans [Limit not valid in Global Health; records were retained] | 2927 |
| 39 | limit 38 to yr="1980 - 2016" | 2854 |
| 40 | remove duplicates from 39 | 1958 |
