# Supplementary material for: Risk of childhood mortality associated with death of a mother in low-and-middle-income countries: a systematic review and meta-analysis
Source: BMC Public Health. 2019 Oct 11;19:1281. doi: 10.1186/s12889-019-7316-x (PMC6788023; doi:10.1186/s12889-019-7316-x)
Supplement: Supplementary file 2 — Supportive information on detailed study characteristics, risk of bias assessment, and extracted results of studies included in meta-analysis. (DOCX 66 kb) [file 12889_2019_7316_MOESM2_ESM.docx]

**Additional file 2**

Table S3: Study characteristics

Table S4: Risk of bias assessment

Table S5: Extracted data of eligible studies included in meta-analysis: Relative risks of childhood mortality by age at time of mother’s death compared to children whose mother survived

Table S6: Extracted data of eligible studies included in meta-analysis: Relative risk of childhood mortality by time since mother’s death and age at risk of child death

### Additional file 2: Supportive information on risk of detailed study characteristics, bias assessment, and extracted data

### Table S3: Study characteristics of 15 articles reporting 13 cohorts of 12 included original studies in the systematic review and meta-analysis of the impact of mother’s deaths on childhood mortality, 1980-2017

| **Study** | **Articles** | **Study design** | **Population and database** | **Exposure** | **Comparator** | **Outcome** | **Comments** |
| --- | --- | --- | --- | --- | --- | --- | --- |
| 1 | 1) Finlay 2015 (rural Tanzania, Low-Income) | Cohort | Children born between 1996 and 2012 covered by the Ifakara health and demographic surveillance system (HDSS) and children born between 1999 and 2010 covered by the Rufiji HDSS  N = 77,777  (N used for analyses for maternal death before one year of age) | Death of mother:  when child aged 0-42 days  and child survived their mother  N exposed = 121  when child aged 0-1 year and child survived their mother  N exposed = 248 | Mother alive at end of study period  N = 76,362  for analyses for maternal death before one year of age | Child mortality at ages:  0-30 days  1-6months  6-12 months  1-2 years  2-3 years | Exposures and outcomes derived from information collected from each household village in quarterly censuses  Follow-up from birth until death, migration out of study community or end of study period  HIV infection not mentioned  Cause of maternal death not included in analyses as subject to recall bias (causes were identified by verbal autopsy)  Authors note maternal deaths were rare resulting in a small exposed sample which shrank due to mortality effects in infancy resulting in less stable estimates for survival beyond 2 years  Poison regression used to compare the death rates for different age groups |
| 2 | 2) Moucheraud, 2015 (rural and urban Ethiopia, Low-Income) | Cohort | Children born between 1987-2011 in Butajira district covered by a health and demographic surveillance system  N = 17,993 | Death of mother:  when child aged 0-42 days and child survived their mother  N exposed = 32  when child aged 0-365 days and child survived their mother  N exposed = 60  when aged > 1 year and child survived their mother  N exposed = 907 | Mother alive at end of study period  N unexposed = 16,875 | Child mortality at ages:  0-30 days  1-6months  6-12 months  1-2 years  2-3 years | Exposures and outcomes were derived from monthly household visits (1987-2000) and later (2000-2011) quarterly household visits  Follow-up from birth until death, migration out of study community or end of study period  Poison regression used to compare the death rates for different age groups  HIV and anemia could affect both maternal and infant survivals |
| 3 | 3) Houle 2015 (rural South Africa, Upper-Middle-Income)* | Cohort | Children aged up to the age of 10 years born during 1992-2013 in 27 villages covered by the Agincourt health and socio-demographic surveillance system (HDSS)  N = 70,418  *Subgroup*  Children born between 2001 – 2013 with SES data for family | Mother died:  Within 43-365 days since most recent birth  N exposed = 100  More than 1 year after most recent birth  N exposed = 597  Analysis for children who survived their mother | Mother alive throughout follow-up period  N = 69,637 | Child mortality at age 43 days - 10 years | Exposures and outcomes derived from annual interview of most knowledgeable person in each household  Follow-up until death or censoring ie exit from the population  Almost a quarter of women were HIV-positive  Causes of maternal and child deaths were identified by verbal autopsy using a model with high HIV prevalence and low malaria prevalence  Relative risk regression analyses |
|  | 4) Clark 2013(rural South Africa, Upper-Middle-Income)* |  | *Subgroup*  Children aged up to 5 years born between 1994-2008 in 21 villages covered by the Agincourt HDSS  N = 41,584 | Mother died due to all causes except AIDS/TB and accidents when child aged 0-5 years  N = 950 | Mother alive  N = 40,634 | Child mortality at age 0-5 years | Multi-level logistic regression model |
|  |  |  |  | Mother died:  < 2 months ago  3-5 months ago  6 or more months ago | Mother alive and not terminally ill | Child mortality at age 0-5 years |  |
|  | 5) Sartorius 2010 (rural South Africa, Upper-Middle-Income)* |  | *Subgroup*  Children resident in 21 villages in Agincourt HDSS between 1992 – 2007 aged 0-1 years  N = 31,804 | Mother died when child aged 0-1 years | Mother alive at end of follow-up | Child mortality at age 0-1 years | Multivariate Bayesian negative binomial model |
|  | 6) Sartorius 2011 (rural South Africa, Upper-Middle-Income* |  | *Subgroup*  Children resident in 21 villages in Agincourt HDSS between 1992 - 2007 aged 1 - 4 years  N = 46,675 | Mother died when child aged 1-4 years | Mother alive at end of follow-up | Child mortality at age 1-4 years  Mean follow-up = 23.7 months | Multivariate geostatistical-temporal model |
| 4 | 7) Saleem, 2014 (India (lower-middle income), Pakistan (lower-middle income), Argentina (upper-middle income), Kenya low income), Zambia (low income), Guatemala (low-income) | Cohort | Pregnancies between 2010 and 2012 in 7 sites covering 124 rural or semi-urban communities  N = 214,566 | Death of mother when child aged 0-42 days  N exposed = 227 | Mother alive six weeks after delivery  N unexposed = 214,221 | Child mortality at ages:  0-6 days  0-27 days | Exposures and outcomes were collected by trained registry administrators or study coordinators from community elders, birth attendant, other health care workers and/or hospital birth records.  Follow-up from birth to six weeks after delivery  Data pooled from six countries  Causes of maternal deaths were ascertained by the supervising physician at each study site.  Risk ratios calculated |
| 5 | 8) Ronsmans, 2010 (rural Bangladesh - lower-middle income) | Cohort | Children born between 1982 and 2005 in Matlab area covered by a health and demographic surveillance system (HDSS)  N = 144,861 | Death of mother when child aged 0-10 years  N exposed = 1,385 (1%)  Analysis for children who survived their mother  N = 158 | Mother alive  N unexposed = 143,473 | Child mortality at ages:  0-1 month  1-5months  6-11 months  12-23 months  36-47 months  48-59 months  60-119 months | Exposures and outcomes were derived from fortnightly and later 2-monthly two months household visits by community health workers  Follow-up from birth to age 120 months or until they died, mother immigrated or study end with follow-up for children whose mothers died divided into 2 periods: when mother alive and when mother dead  Causes of death were assigned by health assistants (1982-1987) and later (1987-2005) by physicians  Poisson regression was used to compare death rates |
| 6 | 9) Becher 2004 (Burkina Faso – low income) | Cohort | Children born between 1st January 1993 and 31st December 1999 in 39 villages in Nouna Health District covered by the demographic surveillance system (DSS) of the Centre de Research en Sante de Nouna (CRSN)  N = 10,122 | Death of mother when child aged 0-3 years  N = 42 | Mother alive    N = 9,396 | Child mortality at ages:  0-1 years  1-5 years  Mean follow-up = 2.6 years | Exposures and outcomes derived from routinely collected information from village informants and 3 censuses  Follow-up from birth until death, 5 years of age, immigration or 31/12/1999.  HIV was not a major cause of death – main causes of childhood death were diarrhoea, malaria and respiratory infections.  Data presented for exposure of maternal death before child aged 3 years.  Assume risk ratios reported are for exposure mother died before age 3  Proportional hazards model in which vital status of mother entered as a time dependent variable |
| 7 | 10) Ng'weshemi, 2003  (rural Tanzania – low income) | Cohort | Children born between 1994-2001 in 6 villages and a semi-urban settlement in Magu district covered by a demographic surveillance system.  N = 6049  5% of children were born to HIV-positive mothers | Mother died when child aged 0-12 months  Analysis for children who survived their mother | Mother alive and not terminally ill | Child mortality at age 0-12 months | Exposures and outcomes were derived from routine 6 monthly household visits with standardized questionnaire interviews, 3 HIV serosurveys and antenatal clinic surveillance  Follow-up from birth until death, migration or study end  Comparator used in multiple regression analyses included children whose mothers had died over 1 year ago |
| 8 | 11) Nakiyingi, 2003  (Uganda – low income) | Cohort | Children born between 1989 and 2000 in 15 villages in Masake District included in a longitudinal cohort study (1989-2000) by the Medical Research Council (MRC) Program on AIDS  N = 3,727 | Mother died:  < 1 year ago  N exposed = 58  > 1 year ago  N exposed = 43  Analysis for children who survived their mother | Mother alive and not terminally ill  N = 3,712 | Child mortality at age 0-11 years  Mean follow-up = 3.8 years | Exposures and outcomes derived from routinely collected information from local leaders and annual censuses  Follow-up from birth until death, immigration from study area or until end of study period (2000)  6% of children had mother who was HIV-positive prior to birth  Multivariate hazard analysis presented used non relevant comparator of mother alive or mother died over 1 year ago |
| 9 | 12) Katz, 2003 (rural Nepal – low income) | Cohort | Singleton infants born alive between 1994 and 1997 to women recruited to a randomized community trial in 270 communities in Sarlahi district of a nutritional supplement for childbearing women.  N = 15,469 | Mother died when child aged:  0-7 days  0-28 days  0-24 weeks  and child survived their mother | Mother alive 24 weeks after delivery  N = 14,093 | Child mortality at ages:  0-7 days  8-28 days  4-24 weeks | Exposures and outcomes were derived from household weekly visits by nutrition supplement distributors as part of the community trial.  Follow-up of children was from birth to 24 weeks of age or death  More than 95% women delivered at home  Logistic regression models were used to compare the impact of maternal death on child mortality |
| 10 | 13) Masmas, 2004  (rural Guinea-Bissau – low income)# | Cohort | Selected cohort of children under the age of 5 born to women resident between 1990-1996 in 5 rural regions covered by a demographic surveillance system.  N = 633 | Mother died before child aged 5 years and child survived their mother  N = 128 | Mother was alive at end of follow-up matched by age, gender and area at the time of maternal death to orphans (4 per orphan)  N = 505 | Child mortality at age 0-12 years | Exposures derived from 6-monthly visits to register births and survival of women and children  Outcome determined on interview with the closest relative of the child  Follow–up from mother’s death until death or June 1998  Cox proportional hazards model using age as dependent variable |
| 10 | 13) Masmas, 2004  (urban Guinea-Bissau – low income)# | Cohort | Selected cohort of children under the age of 10 born to women resident between 1990-1997 in 4 districts of Bissau covered by a demographic surveillance system.  N = 494 | Mother died before child aged 10 years and child survived their mother  N = 192 | Mother was alive at end of follow-up matched by age, gender and area at the time of maternal death to orphans (2 per orphan)  N = 302 | Child mortality at age 0-18 years | Exposures derived from regular census  Outcome determined on interview with the closest relative of the child  Follow–up from mother’s death until death or June 1998  Cox proportional hazards model using age as dependent variable |
| 11 | 14) Scott, 2017 (rural and urban Gambia – low income) | Cohort | Children born between 1st April 1989 and 31st December 2014 were linked with their maternal survival status and were followed up until ten years of age under the Farafenni Health and Demographic surveillance system (FHDSS) covered 42 rural villages, a small town and surrounding areas; N=28,502 | Deaths of mother before child reached 10 years  N = 380 | Mother survived  N = 28,122 | Child mortality rates per 1,000 child-years  Overall and age-specific – death rates up to >= 2 years | Exposures and outcomes derived from information collected from each household in quarterly censuses  Follow-up from birth until death, age 10 years, last follow-up visit, or date mother left survey area  HIV infection prevalence <5% 1995-2014 in Gambia  Cox proportional hazards model for different age groups  No adjusted for malaria, HIV/TB, SES, maternal education, … |
| 12 | 15) Anderson, 2007 (rural Haiti – low income) | Nested case-control | Children (index and non-index) of 78 died mothers (cases) and 156 alive mothers (matched control) between 1997 and 1999 in Haiti covered by the Health Information System (HIS) of Haitian Health Foundation (HHF) | Death of mother during pregnancy and 6 weeks after child births.  N exposed=65 | Mother survived through the study  N unexposed = 167 | Child mortality (under 12 years). | Exposures and outcomes were derived from every five-year full censuses and monthly updated.  Additional demographic information was obtained from interviewed with mothers for control and family members for cases  Odd ratio was used to compare the impact of maternal death on child survival |

(*) These 4 articles analyzed 4 different datasets of one original study ( the Agincourt health and socio-demographic surveillance system (HDSS)

(#)The Articles 13 reported 2 different cohorts (rural and urban)

### Table S4: Risk of bias of the 16 data sets of 13 cohorts reporting in 15 articles of 12 studies

| **Assessing items** | **Quality category** | **N (%)** |
| --- | --- | --- |
| 1 | Selection of the exposed and non-exposed cohorts  Low risk of bias  Moderate risk of bias  High risk of bias | 16 (100)  0 (0)  0 (0) |
| 2 | Measurement of exposure  Low risk of bias  Moderate risk of bias  High risk of bias | 0 (0)  16 (100.0)  0 (0) |
| 3 | Measurement of outcome  Low risk of bias  Moderate risk of bias  High risk of bias | 1 (6.25)  15 (93.75)  0 (0) |
| 4 | Was outcome of interest absent at the time to which the exposure refers?  Low risk of bias  Moderate risk of bias  High risk of bias | 2 (12.5)  14 (87.5)  0 (0) |
| 5 | Was follow-up long enough for outcome to occur?  Low risk of bias  High risk of bias | 16 (100)  0 (0) |
| 6 | Participation rate  Low risk of bias  Moderate risk of bias  High risk of bias | 16 (100)  0 (0)  0 (0) |
| 7 | Completeness of follow-up  Low risk of bias  Moderate risk of bias  High risk of bias | 12 (75.0)  3 (18.75)  1(6.25) |
| 8 | Accuracy of dates of outcome or censoring  Low risk of bias  High risk of bias | 16(100)  0 (0) |
| 9 | Difference in follow-up between exposed and non-exposed  Low risk of bias  Moderate risk of bias  High risk of bias | 15 (93.75)  0 (0)  1 (6.25) |
| 10 | Difference in missing data for exposure between those with or without the outcome  Low risk of bias  Moderate risk of bias  High risk of bias | 16 (100.0)  0 (0)  0 (0) |
| 11 | Comparability of exposed and non-exposed cohorts with respect to potentially important confounding variables  Low risk of bias  Moderate risk of bias  High risk of bias | 6 (37.5)  4 (25.0)  6 (37.5) |
| 12 | Covariates are appropriately included in statistical analysis models  Low risk of bias  High risk of bias | 16 (100.0)  0(0) |

### Table S5: Relative risks of childhood mortality by age at time of mother’s death compared to children whose mother survived - Results of individual studies included in meta-analysis

| **Study** | | **Exposure**  **Maternal death** | | **Outcome Child mortality/survival** | | **Results** | | **Effect size (95% CI)** |
| --- | --- | --- | --- | --- | --- | --- | --- | --- |
|  | | **Time of maternal death** | **Cause of maternal death** | **Mortality**  **Age of child at death** | **Metric** | **Exposed** | **Non-exposed** |  |
| ***Exposure = mother died when child aged 0-7 days*** | | | | | | | | |
| Katz, 2003  (rural Nepal) | | Mother died when child aged 0-**7** days | All causes | 0-7 days | Deaths per 1000 live births (n deaths) | 234.0 (11) | 27.7 (398) | OR = 6.43 (2.35-17.56)^h^ |
| Scott, 2017 (rural and urban Gambia) | | Mother died when child aged 0-7 days | All causes | 0-7 days | Deaths per 1,000 child year (95%CI), n deaths | 8,222.23 (3,085.95-21,907.37), 4 | 645.24 (581.06-716.51), 350 | HR = 3.05 (1.12-8.28)^l^ |
| ***Exposure = mother died when child aged 0-28 days*** | | | | | | | | |
| Katz, 2003  (rural Nepal) | | Mother died when child aged 0-**28** days | All causes | 8-28 days | Deaths per 1000 live births (n deaths) | 179.5 (7) | 15.5 (230) | OR = 11.73 (3.82-36.00) ^h^ |
| Ronsmans, 2010 (Bangladesh) | | Mother died when child aged 0-1 month | All causes | 0-1 month | Deaths per 1,000,000 child-days (deaths n) | 1504.1 (37) | 171.5 (6681) | RR = 8.35 (5.73-12.18)^k^ |
| ***Exposure = mother died when child aged 0-42 days*** | | | | | | | | |
| Finlay, 2015 (rural Tanzania) | Mother died when child aged 0-**42 days** | | All causes | < 1 month | Deaths per 100,000 child-days (n deaths) | 831.21 (15) | 82.74 (1,842) | RR = 6.47 (3.25 - 12.87)^aa^ |
|  |  | |  | 1-6 months | Deaths per 100,000 child-days (n deaths) | 251.45 (19) | 10.45 (1,136) | RR = 20.68 (12.71-33.64)^aa^ |
|  |  | |  | 6-12 months | Deaths per 100,000 child-days (n deaths) | 28.89 (2) | 9.38 (1,117) | RR = 2.81 (0.74 - 10.73) ^aa^ |
|  |  | |  | 12-24 months | Deaths per 100,000 child-days (n deaths) | 30.35 (3) | 4.57 (957) | RR = 6.86 (2.19 - 21.45) ^aa^ |
|  |  | |  | 24-36 months | Deaths per 100,000 child-days (n deaths) | 0 (0) | 2.89 (506) | - |
| Moucheraud, 2015 (rural & urban Ethiopia) | Mother died when child aged **0-42 days** | | All causes | < 1 month | Deaths per 100,000 child-days (n deaths) | 4210.53 (20) | 81.92 (433) | RR = 57.24 (25.31-129.49)^j^ |
|  |  | |  | 1-6 months | Deaths per 100,000 child-days (n deaths) | 565.50 (6) | 8.17 (218) | RR = 80.38 (21.93-294.59) ^j^ |
|  |  | |  | 6-12 months | Deaths per 100,000 child-days (n deaths) | 0 | 4.58 (142) | - |
|  |  | |  | 12-24 months | Deaths per 100,000 child-days (n deaths) | 0 | 2.94 (176) | - |
|  |  | |  | 24-36 months | Deaths per 100,000 child-days (n deaths) | 0 | 1.73 (99) | - |
| Saleem, 2014 (semi-urban or rural India, Pakistan, Argentina, Kenya, Zambia, Guatemala) | Mother died when child aged 0-42 days | | All causes | 0-7 days | Deaths per 1,000 live births | 89.9 (31) | 19.3 (4139) | RR = 3.94 (2.74-5.65)* |
|  |  | |  | 0-27 days | Deaths per 1,000 live births | 121.7 (42) | 24.0 (5150) | RR = 7.36 (5.54-9.77)* |
| Masmas, 2004[26]  (rural Guinea-Bissau) | Mother died when child aged 0-42 days | | All causes | 0-12 years | Deaths (n/N) | 22/33 | 28/130 | MR = 3.96 (2.17-7.22)^gg^ |
| Masmas, 2004  (urban Guinea-Bissau) | Mother died when child aged 0-42 days | | All causes | 0-18 years | Deaths (n/N) | 4/11 | 6/21 | MR = 1.39 (0.37-5.20)^g^ |
| ***Exposure = mother died when child aged 0-24 weeks*** | | | | | | | | |
| Katz, 2003  (rural Nepal) | | Mother died when child aged 0-24 weeks | All causes | 4-24 weeks | Deaths per 1000 live births (n deaths) | NR | 19.7 (280) | OR = 51.68 (20.26-131.8 0)^h^ |
| ***Exposure = mother died when child aged 0-12 months*** | | | | | | | | |
| Finlay, 2015 (rural Tanzania) | | Mother died when child aged 0-**365 days** | All causes | < 1 month | Deaths per 100,000 child-days (n deaths) | 831.21 (15) | 83.28 (1,858) | RR = 6.42 (3.22 - 12.79) ^aa^ |
|  | |  |  | 1-6 months | Deaths per 100,000 child-days (n deaths) | 264.84 (30) | 10.61 (1,155) | RR = 22.48 (15.16-33.32)^aa^ |
|  | |  |  | 6-12 months | Deaths per 100,000 child-days (n deaths) | 116.43 (19) | 9.44  (1,125) | RR = 11.60 (7.36 - 18.30) ^aa^ |
|  | |  |  | 12-24 months | Deaths per 100,000 child-days (n deaths) | 46.09 (13) | 4.57 (957) | RR = 9.79 (5.66 - 16.91) ^aa^ |
|  | |  |  | 24-36 months | Deaths per 100,000 child-days (n deaths) | 4.68 (1) | 2.89 (506) | RR = 1.66 (0.23 - 11.85) ^aa^ |
|  | |  |  | 36-48 months | Deaths per 100,000 child-days | 5.90 (1) | 1.55 (230) | RR = 3.79 (0.54 - 26.73) ^aa^ |
|  | |  |  | 48-60 months | Deaths per 100,000 child-days | (0 ) | 0.81 (102) | - |
| Moucheraud, 2015 (rural & urban Ethiopia) | | Mother died when child aged **0 - 365 days** | All causes | < 1 month | Deaths per 100,000 child-days (n deaths) | 1520.91 (20) | 82.05 (433) | RR = 19.42 (9.24-40.85) ^j^ |
|  | |  |  | 1-6 months | Deaths per 100,000 child-days (n deaths) | 240.48 (12) | 7.96 (212) | RR = 27.96 (11.11-70.39) ^j^ |
|  | |  |  | 6-12 months | Deaths per 100,000 child-days (n deaths) | 108.27 (5) | 4.42 (137) | RR = 19.47 (4.85-78.18) ^j^ |
|  | |  |  | 12-24 months | Deaths per 100,000 child-days (n deaths) | 12.52 (1) | 2.92 (175) | RR = 0 (0 – 0) |
|  | |  |  | 24-36 months | Deaths per 100,000 child-days (n deaths) | - | 1.74 (99) | - |
| Sartorius, 2010 (rural South Africa) | | Mother died when child aged 0-12 months | All causes | 0-12 months |  | NR | NR | IRR = 51.1 (8.5 – 200.8)^e^ |
| Ng'weshemi, 2003  (rural Tanzania) | | Mother died when child aged 0-12 months | All causes | 0-1 year | Deaths per 1000 live births (95% CI) | 553.4 (269.1-874.1) | 84.0  (76.7 – 92.0) | Rate ratio = 6.59 (3.51-9.50)**calculated from Table 2, in Ng'weshemi, 2003 |
| ***Exposure = mother died when child aged 0-35 months*** | | | | | | | | |
| Becher, 2004  (rural Burkina Faso) | | Mother died when child aged 0-35 months | All causes | 0-12 months | Deaths % (n/N) | 21.4 (9/42) | 6.5 (614/9,396) | RR = 15.6 (7.61 - 31.8)^bb^ |
|  | |  |  | 13-60 months | Deaths % (n/N) | 14.3 (6/42) | 6.6 (624/9,396) | RR = 5.35 (1.69 - 16.9)^bb^ |
| ***Exposure = mother died when child aged 0-5 years*** | | | | | | | | |
| Clark, 2013  (rural South Africa) | | Mother died when child aged 0-5 years | All causes except AIDS/TB & accidents | 0-60 months | Number of child deaths (excluded HIV/TB related deaths) | 118# | 910# | OR = 3.93 (2.30-6.72) ^d^ |
| Masmas, 2004  (rural Guinea-Bissau) | | Mother died when child aged 0-5 years: | All causes | 0-12 years | Deaths (n/N) | 46/128 | 59/505 | MR = 4.24 (2.78-6.47)^g^ |
| ***Exposure = mother died when child aged 0-10 years*** | | | | | | | | |
| Masmas, 2004  (urban Guinea-Bissau) | | Mother died when child aged 0-10 years: | All causes | 0-18 years | Deaths (n/N) | 18/192 | 15/302 | MR = 2.46 (1.17-5.18)^g^ |
| Ronsmans, 2010 (rural Bangladesh) | | Mother died when child aged 0-10 years | All causes | 1-5 months | Deaths per 1,000,000 child-days (n deaths) | 350.6 (50) | 14.0 (2852) | RR = 27.61 (20.27-37.61)^k^ |
|  | |  |  | 6-11 months | Deaths per 1,000,000 child-days (n deaths) | 92.3 (19) | 5.2 (1199) | RR = 18.74 (11.70-30.01)^k^ |
|  | |  |  | 12-23 months | Deaths per 1,000,000 child-days (n deaths) | 34.9 (21) | 4.3 (1845) | RR = 8.20 (5.34-12.61)^k^ |
|  | |  |  | 24-35 months | Deaths per 1,000,000 child-days (n deaths) | 7.0 (7) | 2.3 (898) | RR = 2.85 (1.35-6.02)^k^ |
|  | |  |  | 36-47 months | Deaths per 1,000,000 child-days (n deaths) | 3.6 (5) | 1.3 (458) | RR= 2.53 (1.05-6.13)^k^ |
|  | |  |  | 48-59 months | Deaths per 1,000,000 child-days (n deaths) | 4.5 (8) | 0.8 (270) | RR = 5.09 (2.52-10.28)^k^ |
|  | |  |  | 60-119 months | Deaths per 1,000,000 child-days (n deaths) | 0.9 (11) | 0.4 (507) | RR = 2.13 (1.11-4.07)^k^ |
| Scott, 2017 (rural and suburban Gambia) | | Mother died when child aged 0-10 years | All causes | 7 days to < 1 month | Deaths per 1,000 child year (95%CI), n deaths | 2,679.35 (1,115.22-56,437.23), 5 | 59.72 (49.41-72.18) ,107 | HR = 6.99 (2.98-16.36)^l^ |
|  | |  |  | 1-5 months | Deaths per 1,000 child year (95%CI), n deaths | 542.24 (258.51-1,137.42), 7 | 27.93 (25.01-31.18), 316 | HR = 4.81 (2.30-10.06)^l^ |
|  | |  |  | 6-11 months | Deaths per 1,000 child year (95%CI), n deaths | 165.53 (53.39-513.24), 3 | 25.70 (23.08-28.63),331 | HR = 1.12 (0.23-5.35)^l^ |
|  | |  |  | 12 – 23 months | Deaths per 1,000 child year (95%CI), n deaths | 98.54 (41.01-236.74), 5 | 19.73 (18.01-21.62), 460 | HR = 3.63 (1.56-8.47)^l^ |
|  | |  |  | 24-119 months | Deaths per 1,000 child year (95%CI), n deaths | 4.83 (2.01-11.59),5 | 6.16 (5.70-6.66), 628 | HR = 0.93 (0.37-2.37)^l^ |
|  | |  |  | 0 to 119 months | Deaths per 1,000 child year (95%CI), n deaths | 25.89 (17.99-37.25)(29) | 14.44 (13.85-15.06), 2,192 | HR = 4.66 (3.15-6.89)^l^ |
| ***Exposure = mother died when child aged 6 weeks up to 6 months*** | | | | | | | | |
| Masmas, 2004  (rural Guinea-Bissau) | | Mother died when child aged 6 weeks-6 months | All causes | 0-12 years | Deaths (n/N) | 10/17 | 12/67 | MR = 5.61 (2.07-15.21)^gg^ |
| Masmas, 2004  (urban Guinea-Bissau) | | Mother died when child aged 6 weeks-6 months | All causes | 0-18 years | Deaths (n/N) | 3/6 | 1/12 | No estimate due to skewed distribution of deaths |
| ***Exposure = mother died when child aged 6-11 months*** | | | | | | | | |
| Masmas, 2004  (rural Guinea-Bissau) | | Mother died when child aged 6-11 months | All causes | 0-12 years | Deaths (n/N) | 4/10 | 5/40 | MR = 4.96 (1.09-22.48)^gg^ |
| Masmas, 2004  (urban Guinea-Bissau) | | Mother died when child aged 6-11 months | All causes | 0-18 years | Deaths (n/N) | 3/7 | 2/13 | MR = 7.29 (0.74-71.67)^g^ |
| ***Exposure = mother died when child aged > 12 months*** | | | | | | | | |
| Masmas, 2004  (rural Guinea-Bissau) | | Mother died when child aged 12-23 months | All causes | 0-12 years | Deaths (n/N) | 7/16 | 5/64 | MR = 9.63 (2.43-38.11)^gg^ |
| Masmas, 2004  (urban Guinea-Bissau) | | Mother died when child aged 12-23 months | All causes | 0-18 years | Deaths (n/N) | 2/15 | 2/28 | MR = 2.01 (0.28-14.31)^g^ |
| Sartorius, 2011  (rural South Africa) | | Mother died when child aged 1-4 years | All causes except AIDS/TB & accidents | 12-59 months | NR | NR | NR | IRR = 5.17 (2.84 – 8.29)^f^ |
| Moucheraud, 2015 (rural & urban Ethiopia) | | Mother died when child aged > 1 year | All causes | NR | Deaths (n/N) | 31/907 | 1509/16875 | NR |
| Masmas, 2004  (rural Guinea-Bissau) | | Mother died when child aged 24-60 months | All causes | 0-12 years | Deaths (n/N) | 3/52 | 9/204 | MR = 1.64 (0.44-6.16)^gg^ |
| Masmas, 2004  (urban Guinea-Bissau) | | Mother died when child aged 24-120 months | All causes | 0-18 years | Deaths (n/N) | 6/153 | 4/228 | MR = 2.13 (0.60-7.56)^g^ |

CI = confidence interval; HIV = human immunodeficiency virus; TB = Tuberculosis;IRR = incidence rate ratio; MR = mortality rate ratio; NR = Not reported; OR = odds ratio; RR = rate/risk ratio; HR = Hazard Ratio;

aa Poisson regression adjusting for child sex, twinship, mother’s age, mother’s education and household wealth

bb Cox proportional hazards model which included factors found to be associated with childhood mortality in this population ie sex, age, year of birth, ethnic group, religion, age of mother at birth of child, season of birth, twin birth, birth order, distance from health centre, time till birth of next sibling, time since last sibling was born and vital status of last sibling

d Multi-level logistic regression model which included sex, age, year, time before and after mother’s death and multiple birth

e Spatio-temporal multivariate model which included year of birth, number of household deaths, previous death of sibling or stillbirth, gender, pregnancy parity

f Spatio-temporal multivariate model which included year, age, paternal death before age 5, number of children in household aged < 5

g Cox proportional hazards model corrected for age, gender, residence and orphan’s age at mother’s death

gg Cox proportional hazards model corrected for age, gender and residence

h Logistic regression model which included maternal age, maternal and paternal education, sex, previous miscarriages, prior child deaths, parity, gestational age

i Adjusted for family and sex

j Adjusted for household wealth, mother’s age, mother’s marital status and mother’s educational attainment

k Adjusted for year of birth, district, sex, gravida, maternal education and age, husband’s SES

l Adjusted for year of birth, rural/urban, mother’s age, birth spacing

* Univariate analyses/ not adjusted for any confounders including age

** Effect estimate calculated from published data - not adjusted for any confounders including age

# Calculated from Table 1 in Clark, 2013

### Table S6: Relative risk of childhood mortality by time since mother died and age at risk of child death - Results from individual studies included in meta-analysis

| **Study** | **Exposure**  **Maternal death** | | **Outcome**  **Child mortality/survival** | | **Results** | | **Effect size (95% CI)** |
| --- | --- | --- | --- | --- | --- | --- | --- |
|  | **Death of a mother by child ages** | **Cause of death of a mother** | **Age of child at death** | **Metric** | **Exposed** | **Non-exposed** |  |
| Clark, 2013  (rural South Africa) | Mother died 0-2 months ago | All causes | 0-60 months | Deaths per 1,000 child-months | 30.5 | 0.9 | OR = 7.01 (3.16-15.56) ^dd^ |
| Clark, 2013  (rural South Africa) | Mother died 3-5 months ago | All causes | 3-60 months | Deaths per 1,000 child-months | 13.6 | 0.9 | OR = 4.03 (1.53-10.58) ^dd^ |
| Masmas, 2004  (rural Guinea-Bissau) | Mother died before child aged 5 years and  died 0-6 months ago | All causes | 0-12 years | Deaths (n) | 34 | NR | MR = 5.59 (3.44-10.26)^g^ |
| Masmas, 2004  (urban Guinea-Bissau) | Mother died before child aged 10 years and  died 0-6 months ago | All causes | 0-18 years | Deaths (n) | 12 | NR | MR = 3.09 (1.27-7.49)^g^ |
| Nakiyingi, 2003  (Uganda) | Mother died 0-12 months ago | All causes | 0-11 years | Deaths per child-years (n deaths) | 0.14 (7) | 0.029 (398) | RR = 4.96 (2.35 – 10.47)* |
| Masmas, 2004  (rural Guinea-Bissau) | Mother died before child aged 5 years and  died > 6 months ago | All causes | 6 months -12 years | Deaths (n) | 12 | NR | MR = 2.56 (1.29-5.09)^g^ |
| Masmas, 2004  (urban Guinea-Bissau) | Mother died before child aged 10 years and  died > 6 months ago | All causes | 6 months -18 years | Deaths (n) | 6 | NR | MR = 1.77 (0.61-5.11)^g^ |
| Clark, 2013  (rural South Africa) | Mother died 6-60 months ago | All causes | 6-60 months | Deaths per 1,000 child-months | 3.0 | 0.9 | OR = 1.59 (0.61-4.15) ^dd^ |
| Nakiyingi, 2003 (Uganda) | Mother died > 12 months ago | All causes | 1-11 years | Deaths per child-years (n deaths) | 0 | 0.029 (398) | No deaths in exposed group |

CI = confidence interval; MR = mortality rate ratio; NR = Not reported; OR = odds ratio; RR = rate/risk ratio

^dd^ Multi-level logistic regression model which included sex, age, year, multiple birth and mother’s cause of death

^g^ Cox proportional hazards model corrected for age, gender, residence and orphan’s age at mother’s death

* Univariate analyses/ not adjusted for any confounders including age
