# Supplementary material for: Risk of childhood mortality associated with death of a mother in low-and-middle-income countries: a systematic review and meta-analysis
Source: BMC Public Health. 2019 Oct 11;19:1281. doi: 10.1186/s12889-019-7316-x (PMC6788023; doi:10.1186/s12889-019-7316-x)
Supplement: Supplementary file 3 — Supportive information on additional analyses and sensitivity analysis. (DOCX 5610 kb) [file 12889_2019_7316_MOESM3_ESM.docx]

**Additional file 3: Supportive information for sub-group analysis and sensitivity analyses**

**S7 Sub-group analysis**

**Child mortality by child age at mother’s death**

### Figure S7A: Mortality risk for children whose mother died at multiple discrete sequential intervals of child’s age


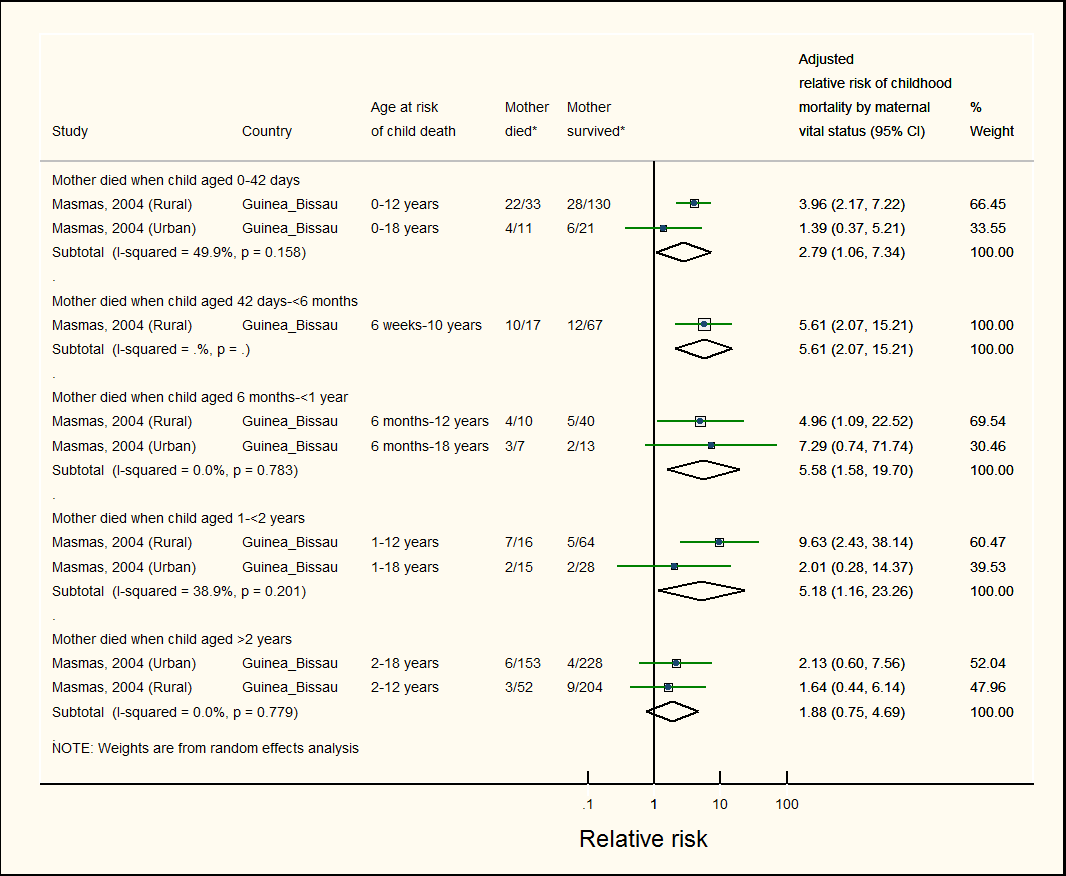


Note: The sizes of boxes on study-specific 95% confidence intervals are proportional to % weight.

Diamond symbols represent the 95% confidence interval for the pooled estimate of each sub-group

(*) Numbers represent child deaths/total children in mother died (exposed) and mother survived (unexposed) groups. These numbers were extracted from the studies but not use to estimate effect sizes. The effect estimates were extracted from the study reports and were usually estimated from multivariate regression models.

**Childhood mortality risk by timing period between mother’s death and child death**

### Figure S7B: Mortality risk for children stratified by time since mother’s death


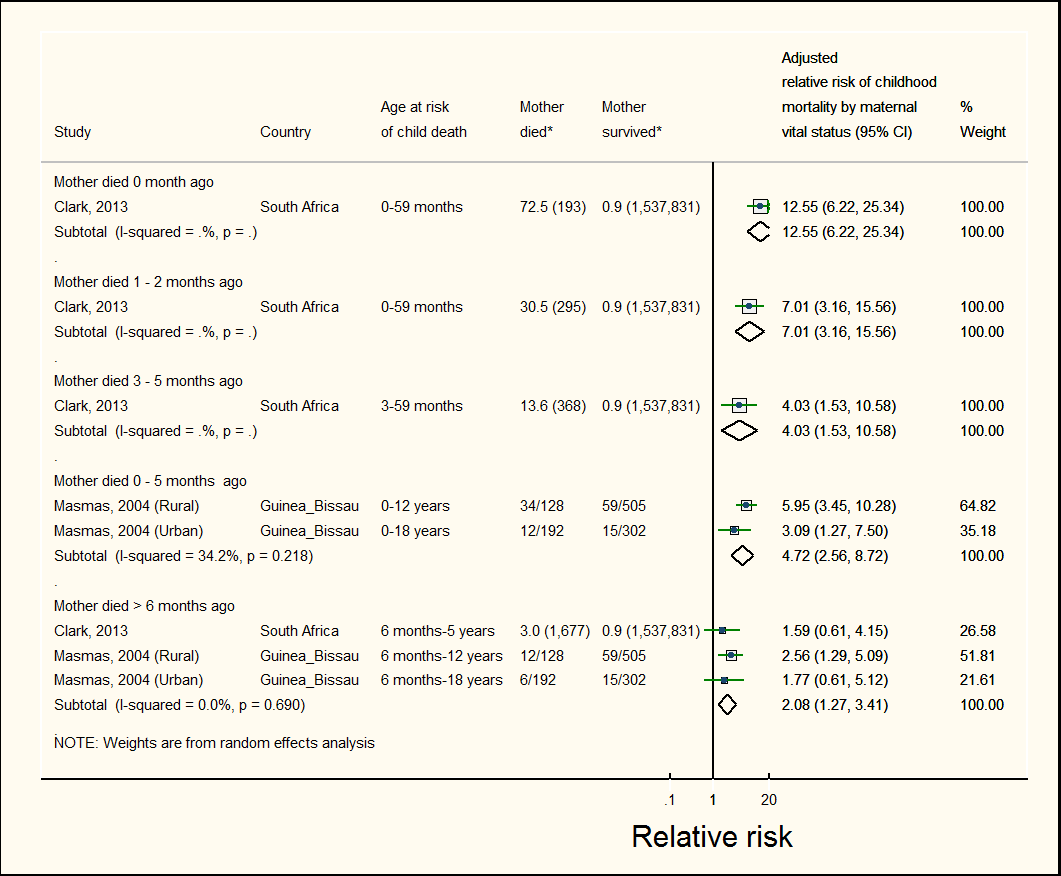


Note: The sizes of boxes on study-specific 95% confidence intervals are proportional to % weight.

Diamond symbols represent the 95% confidence interval for the pooled estimate of each sub-group

(*) Numbers represent mortality rates(number of child-month) (Clark, 2013) or numbers of child deaths/total children (Masmas, 2004) in mother died (exposed) and mother survived (unexposed) groups. These numbers were extracted from the studies but not directly use to estimate effect sizes. The effect estimates were extracted from the study reports and were usually estimated from multivariate regression models

Clark, 2003 reported mortality rate per 1,000 child-months;

Masmas,2004 reported number of child deaths per total number of children

**S8 Sensitivity analysis**

It is acknowledged that some infectious diseases, particularly HIV infection, could be potential confounders of the association between mother’s death and childhood mortality. It has been reported that death of a mother increased substantially in sub-Saharan Africa during the 1990s, as a result of the HIV pandemic.[[16](#_ENREF_16)] It has also been reported that children who were born to HIV-infected mothers are more likely to die than those born to HIV-uninfected mothers.[[17](#_ENREF_17)] Therefore, we pre-specified a criterion to exclude any studies that only reported the results for children of HIV-infected mothers.

### Figure S8A: Relative risk of childhood mortality for children whose mothers died when child was aged up to 42 days compared to children whose mother survived - Sensitivity analysis by stratifying for HIV prevalence of study settings


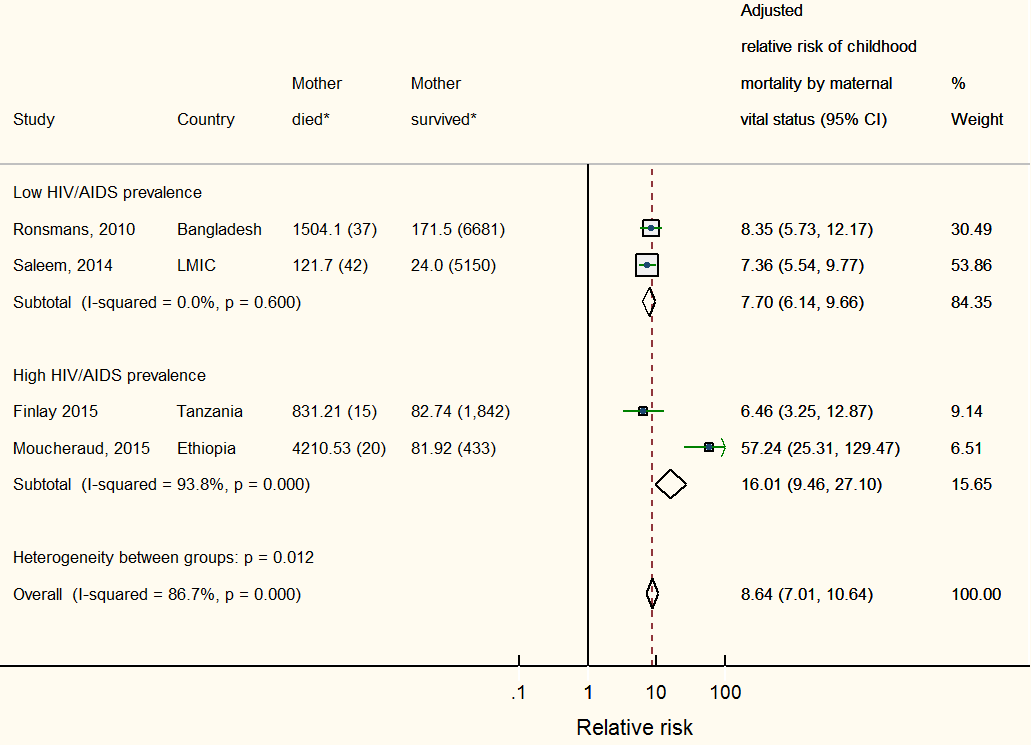


Notes: Result from fixed-effects meta-analysis

The sizes of boxes on study-specific 95% confidence intervals are proportional to % weight.

Diamond symbols represent the 95% confidence interval for the pooled estimate of each sub-group

(*) Numbers represent mortality rates (number of child deaths) in mother died (exposed) and mother survived (unexposed) groups. These numbers were extracted from the studies but not directly use to estimate effect sizes. The effect estimates were extracted from the study reports and were usually estimated from multivariate regression models

Ronsmans, 2010; Finlay, 2015; Moucheraud, 2015 reported deaths per 100,000 child-days (n deaths)

Saleem, 2014 reported deaths per 1000 live births (n deaths)

### Figure S8B: Relative risk of childhood mortality for children whose mothers died when child was aged up to 42 days compared to children whose mother survived - Sensitivity analysis by stratifying results for HIV prevalence of study settings and excluding Moucheraud, 2015


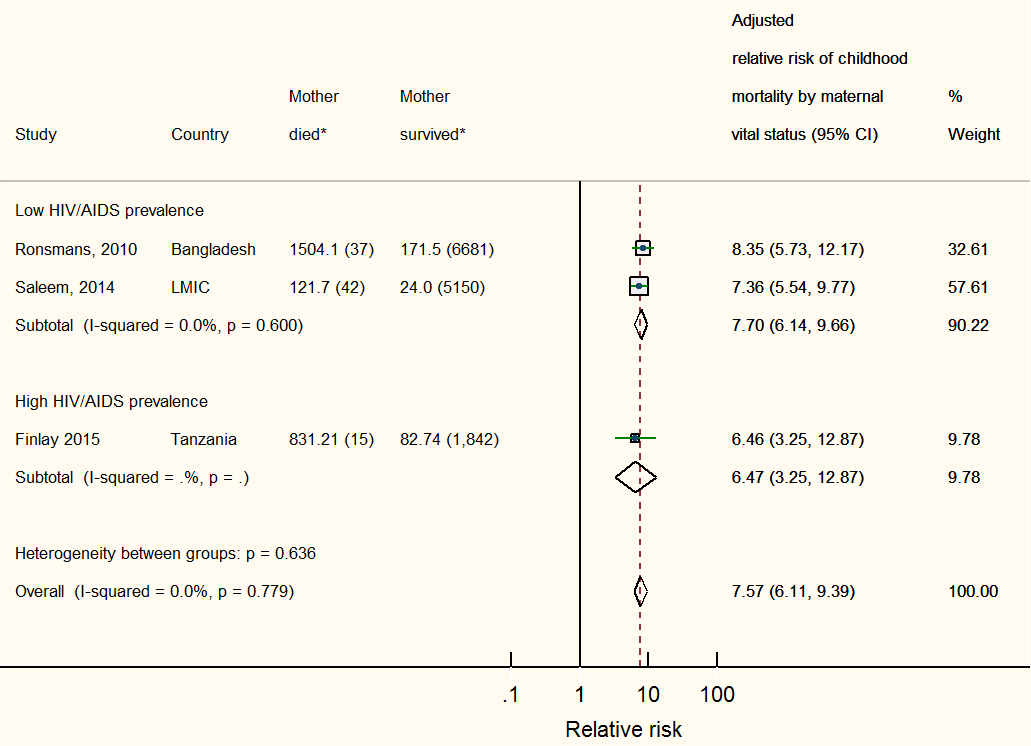


Notes: Result from fixed-effects meta-analysis

The sizes of boxes on study-specific 95% confidence intervals are proportional to % weight.

Diamond symbols represent the 95% confidence interval for the pooled estimate of each sub-group

(*) Numbers represent mortality rates (number of child deaths) in mother died (exposed) and mother survived (unexposed) groups. These numbers were extracted from the studies but not directly use to estimate effect sizes. The effect estimates were extracted from the study reports and were usually estimated from multivariate regression models

Ronsmans, 2010 and Finlay, 2015 reported deaths per 100,000 child-days (n deaths)

Saleem, 2014 reported deaths per 1000 live births (n deaths)

### Figure S8C: Relative risk of childhood mortality for children aged 0-7 days) whose mothers died 0-42 days, age at risk of child death at 0-7 days - Sensitivity analysis stratifying OR (odd ratio) vs. other effect measurements


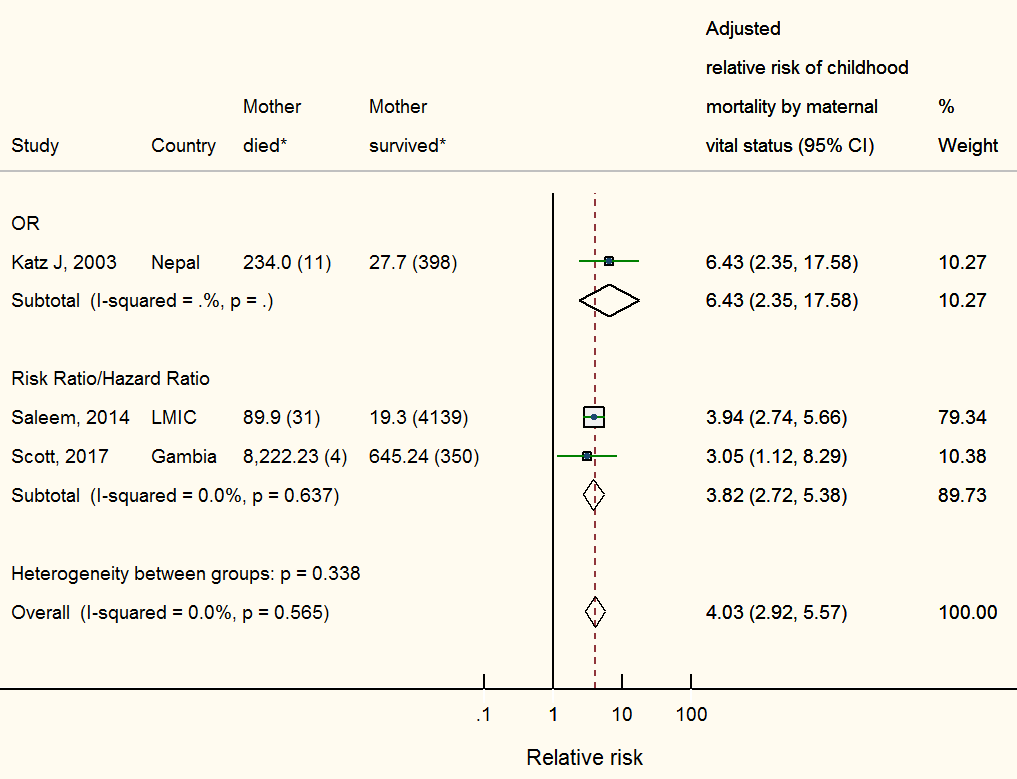


OR: Odd Ratio

Notes: Result from fixed-effects meta-analysis

The sizes of boxes on study-specific 95% confidence intervals are proportional to % weight.

Diamond symbols represent the 95% confidence interval for the pooled estimate of each sub-group

(*) Numbers represent mortality rates (number of child deaths) in mother died (exposed) and mother survived (unexposed) groups. These numbers were extracted from the studies but not directly use to estimate effect sizes. The effect estimates were extracted from the study reports and were usually estimated from multivariate regression models

Katz, 2003 and Saleem, 2014 reported deaths per 1000 live births (n deaths)

Scott, 2017 reported mortality rate per 1,000 child years

### Figure S8D: Relative risk of childhood mortality for children whose mothers died 0-12 months, age at risk of child death at 1-6 months - Sensitivity analysis stratifying OR (odd ratio) vs. other effect measurements


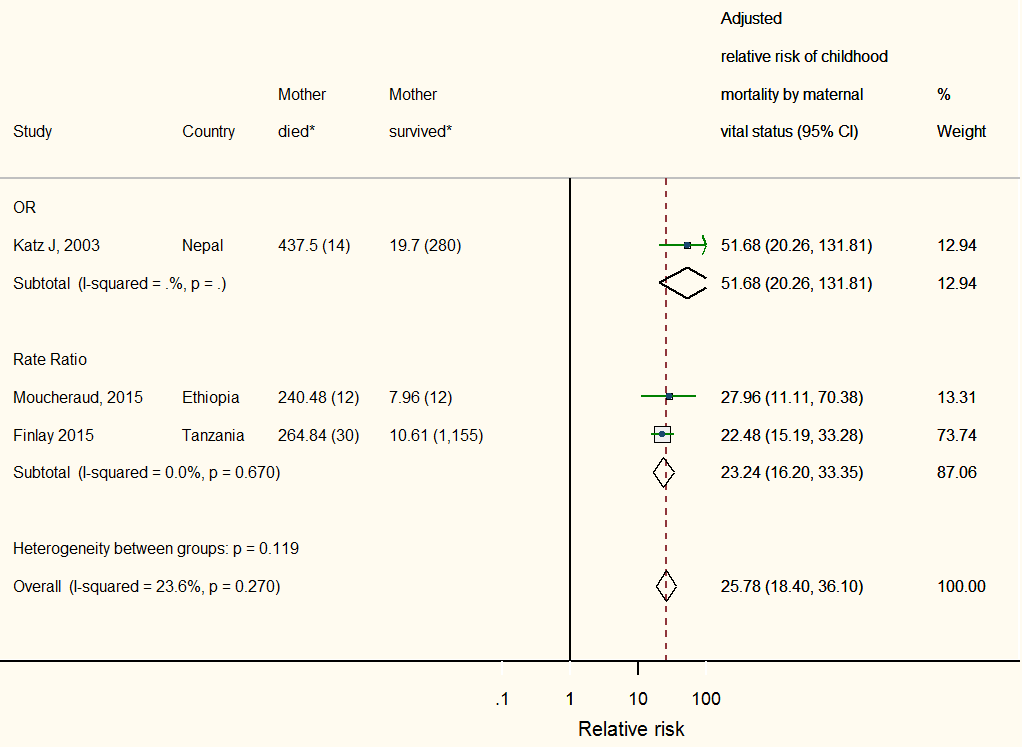


OR: Odd Ratio

Notes: Result from fixed-effects meta-analysis

The sizes of boxes on study-specific 95% confidence intervals are proportional to % weight.

Diamond symbols represent the 95% confidence interval for the pooled estimate of each sub-group

(*) Numbers represent mortality rates (number of child deaths) in mother died (exposed) and mother survived (unexposed) groups. These numbers were extracted from the studies but not directly use to estimate effect sizes. The effect estimates were extracted from the study reports and were usually estimated from multivariate regression models

Katz, 2003 reported deaths per 1000 live births (n deaths)

Finlay, 2015 and Moucheraud, 2015 reported deaths per 100,000 child-days (n deaths)
